# TREM2^+^ and interstitial-like macrophages orchestrate airway inflammation in SARS-CoV-2 infection in rhesus macaques

**DOI:** 10.1038/s41467-023-37425-9

**Published:** 2023-04-06

**Authors:** Amit A. Upadhyay, Elise G. Viox, Timothy N. Hoang, Arun K. Boddapati, Maria Pino, Michelle Y.-H. Lee, Jacqueline Corry, Zachary Strongin, David A. Cowan, Elizabeth N. Beagle, Tristan R. Horton, Sydney Hamilton, Hadj Aoued, Justin L. Harper, Christopher T. Edwards, Kevin Nguyen, Kathryn L. Pellegrini, Gregory K. Tharp, Anne Piantadosi, Rebecca D. Levit, Rama R. Amara, Simon M. Barratt-Boyes, Susan P. Ribeiro, Rafick P. Sekaly, Thomas H. Vanderford, Raymond F. Schinazi, Mirko Paiardini, Steven E. Bosinger

**Affiliations:** 1 Division of Microbiology and Immunology, Emory National Primate Research Center, Emory University, Atlanta, GA USA; 2 Emory NPRC Genomics Core Laboratory, Emory National Primate Research Center, Emory University, Atlanta, GA USA; 3 Department of Infectious Diseases and Microbiology, University of Pittsburgh, Pittsburgh, PA USA; 4 Department of Pathology and Laboratory Medicine, School of Medicine, Emory University, Atlanta, GA USA; 5 Department of Medicine, School of Medicine, Emory University, Atlanta, GA USA; 6 Department of Microbiology and Immunology, Emory School of Medicine, Emory University, Atlanta, GA USA; 7 Department of Immunology, University of Pittsburgh, Pittsburgh, PA USA; 8 Department of Pediatrics, School of Medicine, Emory University and Children’s Healthcare of Atlanta, Atlanta, GA USA

**Keywords:** SARS-CoV-2, Viral pathogenesis, Infection, Viral infection

## Abstract

The immunopathological mechanisms driving the development of severe COVID-19 remain poorly defined. Here, we utilize a rhesus macaque model of acute SARS-CoV-2 infection to delineate perturbations in the innate immune system. SARS-CoV-2 initiates a rapid infiltration of plasmacytoid dendritic cells into the lower airway, commensurate with IFNA production, natural killer cell activation, and a significant increase of blood CD14^-^CD16^+^ monocytes. To dissect the contribution of lung myeloid subsets to airway inflammation, we generate a longitudinal scRNA-Seq dataset of airway cells, and map these subsets to corresponding populations in the human lung. SARS-CoV-2 infection elicits a rapid recruitment of two macrophage subsets: CD163^+^MRC1^-^, and TREM2^+^ populations that are the predominant source of inflammatory cytokines. Treatment with baricitinib (Olumiant®), a JAK1/2 inhibitor is effective in eliminating the influx of non-alveolar macrophages, with a reduction of inflammatory cytokines. This study delineates the major lung macrophage subsets driving airway inflammation during SARS-CoV-2 infection.

## Abstract (web summary)

‘The induction and coordination of immune cells in response to SARS-CoV-2 infection are critical in the immunopathology of COVID-19. Here the authors use a rhesus macaque model of SARS-CoV-2 infection and show key populations of macrophage drive the inflammatory cytokine production in the alveolar space’.

## Introduction

The COVID-19 pandemic began with a series of reports of localized outbreaks of pneumonia caused by a novel coronavirus, SARS-CoV-2, in Wuhan, China in December 2019^1,2^. As of early 2023, there have been over 672 million documented infections, and nearly 7 million fatalities attributed to sequelae of COVID-19. The rapid development and availability of effective vaccines^3–5^ against SARS-CoV-2 infection has provided much needed optimism that infection rates will decline and that the containment of the virus at the population level is possible. Despite these landmark achievements, continued research efforts are essential to safeguard against potential breakthrough variants, to develop therapies for those afflicted while the vaccine rollout continues, and to prevent or minimize the impact of future viral outbreaks. In this light, basic research into the innate and adaptive immune responses to SARS-CoV-2 continues to be critical for informing vaccine and therapeutic approaches directed at ending the COVID-19 pandemic or at decreasing mortality.

Since the emergence of the COVID-19 pandemic, research into the virology, immune responses and pathogenesis of SARS-CoV-2 infection has amassed at an unprecedented rate, and numerous hypotheses have arisen to explain the underlying mechanisms of severe COVID-19. Of these, the concepts that have accumulated the most supporting evidence are: (1) evasion or impairment of early Type I interferon (IFN) responses^6^, (2) vascular complications arising from hypercoagulability syndromes^7^, and (3) perturbations of the granulocyte and myeloid compartments in the lower airway and blood manifesting in inflammatory cytokine production^8,9^. Immunologically, severe disease in COVID-19 patients has been associated with a widespread increase in levels of inflammatory mediators (e.g., CXCL10, IL-6, and TNFα) in plasma and bronchoalveolar lavage (BAL) fluid in what is commonly referred to as a “cytokine storm”^10^, and an expansion of macrophages, neutrophils and lymphocytes in the lower airway^8^. Despite this impressive accruement of data, the precise early immunological events and immune cell infiltration that drive inflammation in the lower airway remain uncharacterized.

Non-human primate (NHP) models of SARS-CoV-2 infection (primarily macaque species and African green monkeys (AGMs)) have proven to be critical tools, primarily due to the ability to examine early events after infection longitudinally and in tissues not available in most human studies^11^. NHPs support high levels of viral replication in the upper and lower airway^12–14^, share tissue distribution of ACE2 and TMPRSS2 with humans^15^, and have been invaluable pre-clinical models of vaccines^16–18^ and therapeutics^19,20^. Additionally, mild to moderate COVID-19 has been shown to be recapitulated in SARS-CoV-2-infected NHPs^11^ that typically resolve by 10–15 days post infection (dpi)^11,20,21^. Mechanistic studies of SARS-CoV-2 infection in NHPs have utilized a variety of high-throughput techniques and have reported (1) Type I IFN responses are robustly induced in blood and the lower airway very early after infection^20,22^, (2) elevated pro-inflammatory cytokines consistent with the “cytokine storm” seen in humans are detectable in plasma and BAL^23^, (3) vascular pathology and gene expression consistent with hypercoagulability are evident in the lower airways^22^, and (4) increased production of inflammatory cytokines by myeloid origin cells^20,24^.

In the current study, we used SARS-CoV-2 infected rhesus macaques (RMs) to investigate the early inflammatory events occurring in the blood and lower airway using high dimensional flow cytometry, multi-analyte cytokine detection, and bulk and single-cell RNA-Seq (scRNA-Seq). To dissect the role of discrete immune subsets within the myeloid fraction in SARS-CoV-2-driven inflammation, we used two different strategies, employing scRNA-Seq and bulk-RNA-Seq reference datasets to classify the macrophage/monocyte populations and to identify analogous populations in human airway datasets. With this approach, we identified the main subsets of pro-inflammatory macrophages that expand after SARS-CoV-2 infection and are the predominant source of inflammatory cytokines in the airway. We also observed an early induction of plasmacytoid dendritic cells (pDCs) in blood and the lower airway that coincided with the peak of the IFN signaling. Finally, we described that treatment of SARS-CoV-2 infected RMs with baricitinib, a JAK1/2 inhibitor recently demonstrated to reduce hospitalization time and mortality for severe COVID-19 patients^25^, suppressed airway inflammation by abrogating the infiltration of pro-inflammatory macrophages to the alveolar space. Collectively, this study defines the early kinetics of pDC recruitment and Type I IFN responses, and identifies discrete subsets of infiltrating macrophages as the predominant source of pro-inflammatory cytokine production in SARS-CoV-2 infection.

## Results

### Study overview

**Fig. 1:**
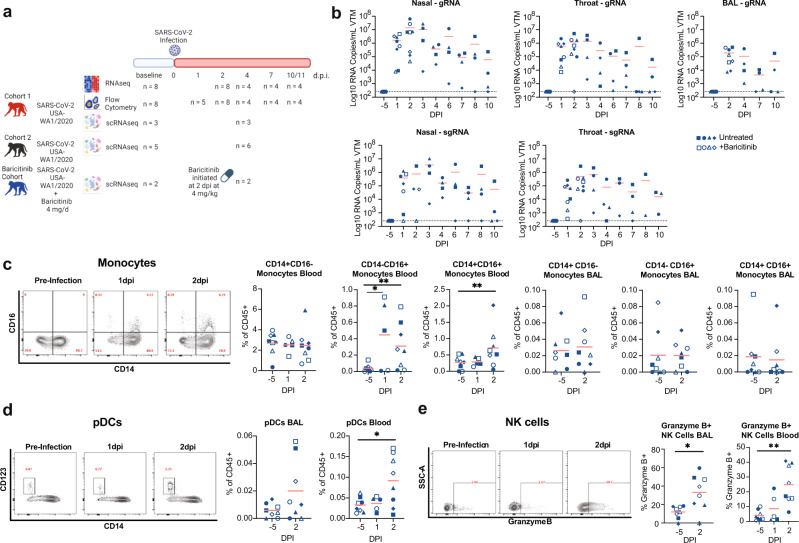
Early expansion of inflammatory cells in the blood following infection with SARS-CoV-2. **a** Study design; RMs were infected intranasally and intratracheally with SARS-CoV-2 and tracked longitudinally (Cohort 1: *n* = 4, baricitnib cohort: *n* = 4, Cohort 2: *n* = 6). Baricitinib was administered daily to 4 RMs (baricitinib cohort) starting at 2 dpi. The 4 RMs  from Cohort 1 and 6 RMs from Cohort 2 were untreated. (Created with BioRender.com). **b** After SARS-CoV-2 inoculation, nasal, throat, and bronchoalveolar lavages (BAL) were collected and viral loads were quantified by qRT-PCR for total gRNA and sgRNA. **c** Longitudinal levels of monocytes within BAL and blood depicted as a % of CD45+ cells. *p* values: CD14−CD16+ Monocytes Blood: 1 dpi vs. −5 dpi = 0.03 and 2 dpi vs. −5 dpi = 0.004; CD14+CD16+ Monocytes Blood 2 dpi vs. −5 dpi = 0.004. **d** Longitudinal levels of plasmacytoid dendritic cells (pDCs) within BAL and blood depicted as a percentage of CD45+ cells. *p* value for pDCs Blood 2 dpi vs. −5 dpi = 0.02. **e** Longitudinal levels of NK cells expressing Granzyme B in BAL and blood. *p* values for Granzyme B+ NK Cells 2 dpi vs. −5 dpi BAL = 0.01 and blood = 0.008. *n* = 8 RM from Cohort 1 and baricitinib cohort. The red bars represent the mean. Statistical analysis was performed using one-tailed Wilcoxon signed-rank test in Graphpad Prism v7.02 comparing each timepoint to −5 dpi. *p value <0.05, **p value <0.01. Source data (**b**–**e**) are provided as a Source Data file.

An overview of the study design is shown in Fig. 1a. We analyzed three separate cohorts of macaques: Cohort 1, Baricitinib-treated and Cohort 2. For Cohort 1 and Baricitinib-treated, a total of eight RMs (mean age 14 years old; range 11–17 years old) were inoculated intranasally and intratracheally with 1.1 × 10^6^ plaque-forming units (PFU) of SARS-CoV-2 (2019-nCoV/USA-WA1/2020). At 2 dpi, four of the eight animals started receiving baricitinib^20^. For this study, pre-infection baseline and hyperacute time points (1–2 dpi) include *n* = 8 RMs, all untreated, and the remaining longitudinal time-points assessed to determine the pathogenesis of SARS-CoV-2 infection are comprised of *n* = 4 of the RMs that remained untreated. Inoculation with SARS-CoV-2 led to reproducibly high viral titers detectable in the upper and lower airways by genomic and sub-genomic qPCR assays (Fig. 1b). The peak of viremia in the nasal passage, throat and BAL was at 2–4 dpi (Fig. 1b). To increase the power of our scRNA-Seq and flow cytometry experiments, we analyzed an additional six macaques infected with the same dose and strain of SARS-CoV-2 (2019-nCoV/USA-WA1/2020) (mean age 10.5 years old; range 6–19.5 years old), referred to as Cohort 2. Animals from Cohort 2 served as SARS-CoV-2-infected, untreated controls in part of a larger study testing the impact of interferon blockade^26^.

### SARS-CoV-2 induces a robust, but transient, expansion of pDCs during hyperacute infection

To characterize the innate immune response following SARS-CoV-2 infection, we analyzed changes in innate populations using multi-parametric flow cytometry in blood and BAL samples in the first 2 dpi, or “hyperacute” phase of infection (Fig. 1c–e and Supplementary Fig. 1), and over the full course of infection (Supplementary Fig. 2). In blood, we did not observe a significant increase in the proportion of classical monocytes (CD14+CD16−) at 2 dpi (Fig. 1c) nor at extended time-points (Supplementary Fig. 2a, d). Similar to reports in humans^9^, we observed a rapid, but transient, increase in blood CD14−CD16+ and CD14+CD16+ monocytes (Fig. 1c and Supplementary Fig. 2a, d). Using these conventional markers for blood monocyte subsets, we did not observe any significant changes in CD14−CD16+, CD14+CD16+, nor CD14−CD16+ within the BAL (Fig. 1c and Supplementary Fig. 2a).

We observed a significantly elevated level of pDCs in blood at 2 dpi and similarly, a trend of elevated pDCs in BAL samples (Fig. 1d and Supplementary Fig. 2c, f). This expansion was transient, as pDC numbers returned to baseline by 4 dpi. While the overall frequencies of natural killer cells (NK) were not changed in blood or BAL (Supplementary Fig. 2b, e), the fraction and absolute number of Granzyme B + NK cells increased significantly at 2 dpi in blood, from 4 to 25% (Fig. 1e) and remained elevated throughout the course of infection (Supplementary Fig. 2b, e). Similarly, increases in NK cell activation were also observed in the BAL, rising from 12 to 33% at 2 dpi (Fig. 1e), and persisting at this level until the study termination at 10/11 dpi (Supplementary Fig. 2b, e). Collectively, these data indicate that during the hyperacute phase of SARS-CoV-2 infection, there is a significant mobilization of innate immune cells capable of initiating and orchestrating effector responses of the Type I IFN system.

### SARS-CoV-2 infection drives robust, but transient, upregulation of IFN responses in blood and lower airway

**Fig. 2:**
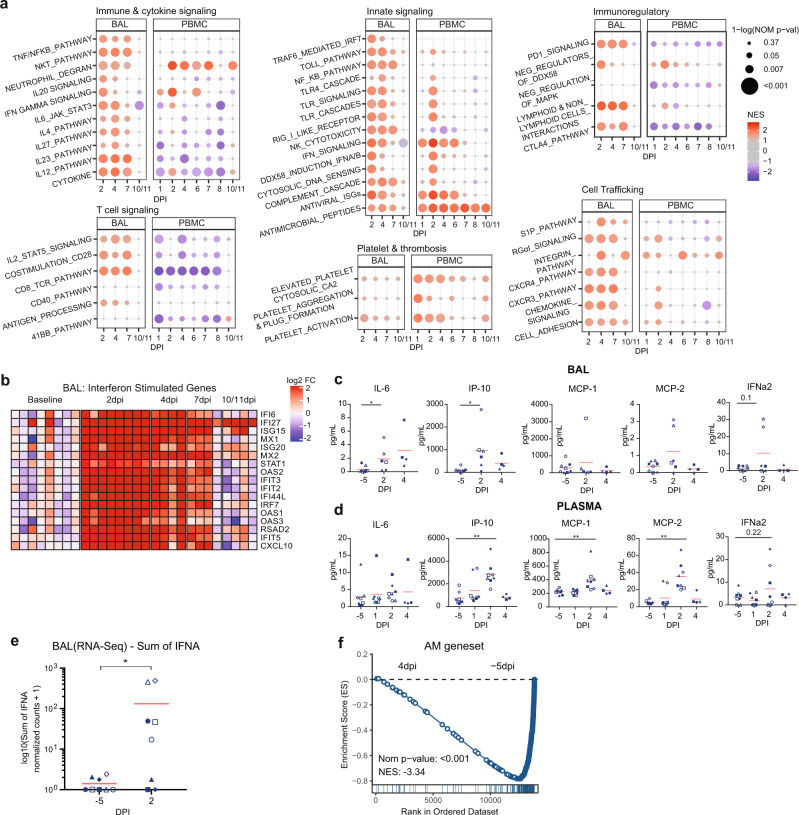
Early pro-inflammatory and ISG response observed in airways and peripheral blood by bulk transcriptomics. *n* = 8 RM from Cohort 1 and baricitinib cohort for −5 dpi and 2 dpi except for (**c**) where *n* = 6 (3 RM Cohort 1 + 3 RM baricitnib cohort) at 2 dpi. *n* = 4 RM from Cohort 1 starting from 4 dpi. **a** Dot plots showing normalized enrichment scores and nominal *p* values for gene sets. Enrichment is indicated by dot color (red: positively enriched vs. −5 dpi; blue: negatively enriched), dot size indicates significance. Normalized enrichment scores and nominal *p* values were determined using GSEA^65^. The exact nominal *p* values are included in Supplementary Data 1. **b** Heatmap of longitudinal responses for the ISG gene set. The color scale indicates log2 expression relative to the median of the −5 dpi samples. **c** Cytokines evaluation (Mesoscale) in BALF *p* values for 2 dpi vs. −5 dpi: IL6 = 0.02 and IP-10 = 0.03 and **d** Plasma; only significant cytokines are shown (*p* values for 2 dpi vs. −5 dpi: IP-10 = 0.008, MCP-1 = 0.008, MCP-2 = 0.004). **e** Sum of normalized expression of all IFNA genes in BAL. *p value = 0.04. **f** GSEA enrichment plot showing negative enrichment for AM gene signature (derived from SingleR) when comparing bulk BAL RNA-Seq samples from 4 dpi to −5 dpi (Nominal *p* value = 0). The red bar represents the mean. Statistical analysis for (**c**)–(**e**) was performed using one-tailed Wilcoxon signed-rank test in R v4.2.2 comparing each timepoint to −5 dpi. *p value <0.05, **p value <0.01. Source data (**c**–**e**) are provided as a Source Data file.

To understand the extent of immunological perturbations induced by SARS-CoV-2 infection, we performed extensive gene expression profiling of PBMC and BAL samples. During the hyperacute phase, the BAL had widespread induction of pathways associated with innate immunity and inflammation (Fig. 2a). Notably, we observed a rapid and robust induction of interferon-stimulated genes (ISGs) in the PBMC and BAL compartments starting at 1 or 2 dpi (Fig. 2b and Supplementary Fig. 3a). The ISG response, although widespread, had largely returned to baseline by 10/11 dpi (Fig. 2b and Supplementary Fig. 3a). We also detected a trend of elevated IFNα protein in 4/6 and 5/8 animals in BAL and plasma, respectively (Fig. 2c, d) and a significant increase in RNA-Seq read counts mapping to IFNA genes at 2 dpi in BAL (Fig. 2e), which coincided with the expansion of pDCs in the airway and blood (Fig. 1d). A significant enrichment of genes representing NK cell cytotoxicity (Fig. 2a) was observed at 2 dpi in BAL, consistent with our observation of elevated Granzyme B + NK cells by flow cytometry (Fig. 1e). Taken together, these data demonstrate the presence of primary cells able to produce Type I IFNs (i.e., pDCs), coincident with detectable IFNA transcripts and protein, and with downstream IFN-induced effector functions (ISGs, NK cell activation) following SAR-CoV-2 infection, and that these responses were transient, having largely subsided by 10/11 dpi.

### SARS-CoV-2 infection drives a shift in airway macrophage populations

We observed that SARS-CoV-2 infection induced significant enrichment of several inflammatory cytokine signaling pathways, namely IFNA, IL4, IL6, IL10, IL12, IL23 and TNF, and the chemokine pathways CXCR4 and CXCR3, in both PBMCs and BAL of RMs, with higher magnitude in the BAL (Fig. 2a and Supplementary Data 1–3). For many of these pathways, we were able to quantify significant increases in the upstream regulator at either the protein, or mRNA level, or both: IL6 protein levels were significantly increased in the BAL fluid (BALF) (Fig. 2c), as were RNA transcripts in BAL (Supplementary Fig. 3b). Similarly, the induction of CXCR3 pathways signaling was consistent with detection of increased IP10/CXCL10 protein in BALF and RNA at 2 dpi in BAL (Fig. 2c and Supplementary Fig. 3b). The appearance of inflammatory pathways in the blood and airway have been reported in a multitude of human studies (reviewed in ref. ^27^). However, we noted that SARS-CoV-2 infection also drove early expression of several immunoregulatory/immunosuppressive pathways in the BAL, namely: PD1 and CTLA4 signaling, and negative regulators of MAP kinase and DDX58/RIG-I signaling (Fig. 2a). Previously, we reported that the myeloid fraction in BAL was primarily responsible for the production of pro-inflammatory mediators, however the specific immunophenotypes were not defined. To further investigate the presence of different macrophage subsets within the lower airway after SARS-CoV-2 infection, we performed GSEA on bulk BAL data using AM gene signature (obtained from SingleR^28^) specific for RM pulmonary macrophages. We observed that genes specific for alveolar macrophages (AMs) were significantly enriched at baseline (−5 dpi) relative to 4 dpi, indicating a downregulation of this gene set after SARS-CoV-2 infection (Fig. 2f). Collectively, these bulk RNA-Seq data indicate a rapid and significant shift in the balance of macrophage populations in the lower airway following SARS-CoV-2 infection.

### SARS-CoV-2 infection induces an influx of two subsets of infiltrating macrophages into the alveolar space

In our prior work in RMs, we demonstrated that cells of myeloid origin were the predominant subset responsible for production of inflammatory cytokines in the lower airway following SARS-CoV-2 infection^20^. While our prior scRNA-Seq analyses determined the majority of cells in the BAL after infection to be of monocyte/macrophage origin, with relatively few neutrophils or granulocytes, the precise immunophenotypes of the myeloid cells driving inflammation in the lower airway have not been precisely delineated.

Cell classification based on cell-surface marker genes is typically problematic in scRNA-Seq data due to gene dropouts inherent to the technology. Accurate classification is further complicated in the rhesus model system, in which genomic references have incomplete annotation, and markers from other model species may not phenocopy. Several significant advances have been made recently elucidating the resident tissue macrophage subsets in the lung and their function during viral infection and inflammation^29–32^. However, analysis of scRNA-Seq data from RM lung suspensions and BAL during steady state condition indicated that several key markers used to differentiate macrophages in the murine lung (e.g., Lyve1) were not expressed at levels sufficient to distinguish populations in the rhesus pulmonary myeloid populations (Supplementary Fig. 7). Therefore, we used two overlapping alternative strategies to accurately classify tissue macrophages and monocyte-derived/infiltrating macrophages in the RM airway after SARS-CoV-2 infection in our scRNA-Seq data. The first strategy was based on using existing lung scRNA-Seq data from uninfected RMs as a reference to map and annotate the BAL cells. We processed lung 10X data from three uninfected RMs (NCBI GEO: GSE149758)^33^ through the Seurat pipeline^34^ and reproduced the four reported macrophage/monocyte subsets: CD163+MRC1+, resembling alveolar macrophages; CD163+MRC1+TREM2+ macrophages, similar to infiltrating monocytes; CD163+MRC1−, similar to interstitial macrophages; and CD16+ non-classical monocytes (Supplementary Fig. 4a–d). We used Seurat to map BAL macrophages/monocytes from SARS-CoV-2 infected RMs and transfer annotations from the lung reference. The second strategy involved using bulk RNA-Seq on sorted AM and IM from the lungs of three uninfected RMs^35^, according to the phenotype defined by Cai et al.^36^, based on expression of CD206/MRC1 and CD163, to annotate cells using SingleR^28^ Supplementary Fig. 4e, f). In total, 2069 genes were found to be differentially expressed between IMs and AMs (FDR < 0.05, fold-change > 2) (Supplementary Fig. 4e). Of note, CX3CR1 was upregulated in the IMs, consistent with both murine and human definitions of this subset (Supplementary Fig. 4f). APOBEC3A, an RNA-editing cytidine deaminase, was also upregulated in IMs along with PTGS2, a pro-inflammatory COX-2 cyclooxygenase enzyme, TIMP1, which enables migration of cells via the breakdown of connective tissue, VCAN, an immunosuppressive regulator, and PDE4B, which regulates expression of TNFα (Supplementary Fig. 4f). We annotated the lung macrophage/monocyte subsets using the bulk sorted AM and IM datasets and found that almost all of CD163+MRC1+ cluster and some CD163+MRC1+TREM2+ cells were annotated as AM and the remaining as IM (Supplementary Fig. 4g). Thus, benchmarking our lung scRNA-Seq based reference against rudimentary bulk transcriptomic signatures demonstrated their accuracy in resolving the AM phenotype from non-AM in steady state conditions.

**Fig. 3:**
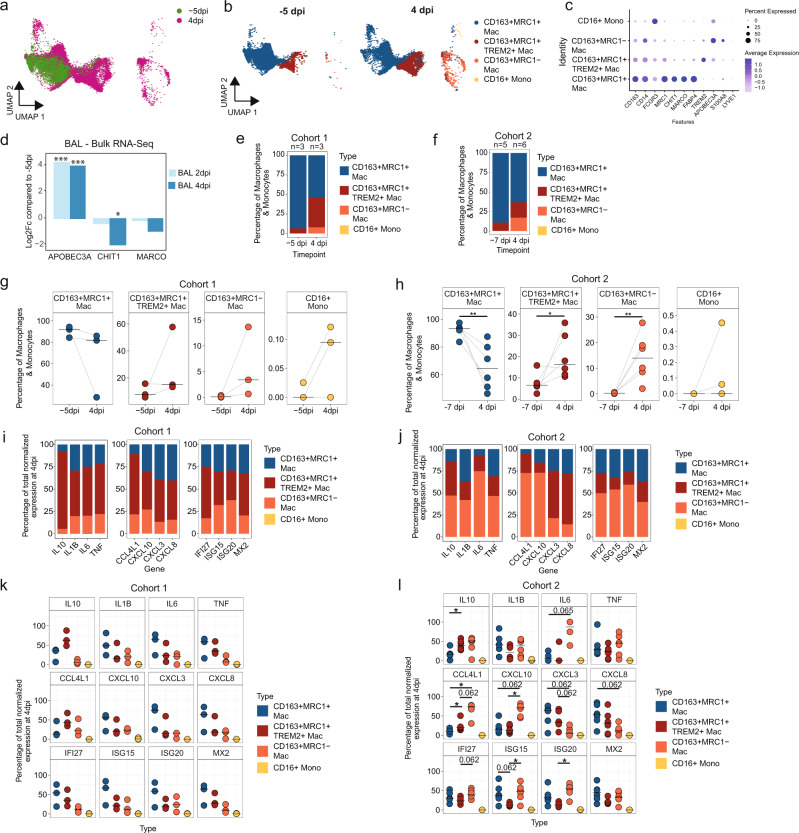
Influx of pro-inflammatory macrophages in BAL. **a** Projection of single-cell macrophages/monocytes from −5 dpi (green) and 4 dpi (magenta) 10X BAL samples obtained from three SARS-CoV-2 infected rhesus macaques (Cohort 1) onto the reference UMAP of lung macrophage/monocytes from uninfected rhesus macaques (NCBI GEO: GSE149758^33^). **b** UMAP projections showing the predicted cell type annotations based on the uninfected lung reference split by time of sample collection (Cohort 1). **c** DotPlots showing the expression of marker genes for the different macrophage/monocyte subsets in SARS-CoV-2 infected BAL samples (Cohort 1). **d** Log2 fold-changes compared to −5 dpi for APOBEC3A, CHIT1 and MARCO in bulk BAL RNA-Seq data (Cohort 1). Significance was determined using DESeq2. The *p* values corrected by the default Benjamini and Hochberg method were used *adj *p* value <0.05, ***adj *p* value <0.001. The exact *p* values are included in Supplementary Data 2. Percentage of a given subset out of all macrophage/monocyte subsets at −5 dpi and 4 dpi from all three animals pooled (Cohort 1) (**e**) and at −7 dpi and 4 dpi from all six animals pooled (Cohort 2) (**f**). Percentage of a given subset out of all macrophage/monocyte subsets for each animal at −5 dpi and 4 dpi from Cohort 1 (*n* = 3) (**g**) and at −7 dpi (*n* = 5) and 4 dpi (*n* = 6) for Cohort 2 (**h**). The black lines indicate the median. *p* values for Cohort 2 (**f**) for 4 dpi vs. −7 dpi: CD163+MRC1+ = 0.009, CD163+MRC1+TREM2+ = 0.03, and CD163+MRC1− = 0.004. Contribution of each macrophage/monocyte subset toward the production of the pro-inflammatory genes and ISG—Cohort 1 pooled (**i**), Cohort 2 pooled (**j**), Cohort 1 individual (**k**) and Cohort 2 individual (**l**). *p* values for (**l**): * = 0.03. The percentage contribution was calculated by dividing the sum of normalized expression of a given gene in a macrophage/monocyte subset by the sum of the normalized expression of the gene in all macrophage/monocyte subsets. **a**–**c**, **e**, **g**, **i**, **k** Cohort 1: *n* = 3 for both −5 dpi and 4 dpi, **d** Cohort 1: *n* = 8 for 2 dpi and *n* = 4 for 4 dpi, **f**, **h**, **j**, **l** Cohort 2: *n* = 5 for −7 dpi and *n* = 6 for 4 dpi. The black lines indicate the median. Statistical analysis was performed using two-tailed Wilcoxon singed rank test for (**g**, **k**, **l**) and two-tailed Mann–Whitney *U* test for (**h**) in R v4.2.2. *p value <0.05, **p value <0.01. Source data (**d**–**l**) are provided as a Source Data file.

We next analyzed changes in the myeloid populations within the BAL of RMs after SARS-CoV-2 infection by applying these signatures to two independent scRNA-Seq datasets from rhesus macaques infected intranasally and intratracheally with 1.1 × 10^6^ PFU of the USA-WA1/2020 strain of SARS-CoV-2: Cohort 1, comprised of a dataset of *n* = 3 (baseline and 4 dpi)^20^ and Cohort 2, comprised of *n* = 5 (baseline) and *n* = 6 (4 dpi)^26^. Using the lung/scRNA-Seq reference, we found that most of the BAL macrophages/monocytes belonged to the AM-like CD163+MRC1+ macrophage subset at −5 dpi along with some cells from the CD163+MRC1+TREM2+ macrophage subset (Fig. 3a, b and Supplementary Fig. 5a). At 4 dpi, there was an influx of both CD163+MRC1+TREM2+ macrophages and the IM-like CD163+MRC1− macrophages with few cells annotated as CD16+ non-classical monocytes. The expression of gene markers such as MARCO, FABP4 and CHIT1 further supported the cell subset annotations (Fig. 3c). We also observed a similar increase in APOBEC3A and decreases in the alveolar macrophage-associated genes MARCO and CHIT1 expression in BAL samples analyzed by bulk RNA-Seq, indicating a loss of CD163+MRC1+ cells (Fig. 3d). By analyzing the sc-RNA-Seq datasets, the percentage total of CD163+MRC1+ macrophages at baseline compared to 4 d.p.i. reduced from 93.3% to 55%, and 89.3% to 63.1% of all macrophages/monocytes in BAL in Cohorts 1 and 2, respectively (Fig. 3e, f). Estimates of cellular frequencies in pooled scRNA-Seq datasets can be driven by unbalanced cell counts from individual samples. To account for this potential bias, we examined the changes in myeloid populations by individual animals. We observed that in Cohort 1, the CD163+MRC+ cells decreased from mean 90.2% (sd = 5.2%) to mean 65.4% (sd = 32%), *p* = ns, and in Cohort 2, they decreased significantly from mean 92% (sd = 5.1%) to mean 65.7% (sd = 16.4%) *p* = 0.0087 (Fig. 3g, h). Conversely, we saw an overall increase in the percentage of CD163+MRC1+TREM2+ macrophages from 6.5 to 36.8% (Cohort 1) and 10.3 to 19.8% (Cohort 2) (Fig. 3e, f). At the individual level, we observed that levels of CD163+MRC1+TREM2+ cells increased from mean 9.7% (sd = 5.3%) to mean 28.6% (sd = 25.2%) in Cohort 1 (*p* = ns), and mean 7.7% (sd = 4.9%) to mean 20.0% (sd = 10.4%) (*p* = 0.03) (Fig. 3g, h). Additionally, we observed increases in the IM-like CD163+MRC1− macrophages: in the pooled scRNA-Seq data from 0.2 to 8% in Cohort 1 and 0.4 to 17% in Cohort 2 (Fig. 3e, f). Considering individual animals, the CD163+MRC1− cells increased from mean 0.15% (sd = 0.19%) to mean 5.9% (sd = 6.9%) in Cohort 1 and significantly from mean 0.28% (sd = 0.18%) to mean 14.2% (sd = 9.1%) (*p* = 0.004) (Fig. 3g, h). Thus, SARS-CoV-2 infection resulted in an influx of monocyte-derived and IM-like macrophages in BAL at 4 dpi.

To further validate our cell classification and support the observation that it is the infiltrating cells that increase in numbers and predominantly produce inflammatory mediators, we used the second strategy of using gene expression of bulk sorted AM and IM cells to classify the BAL macrophages/monocytes. Using this definition, we confirmed that there is an increase in the percentage of non-AM population with a corresponding decrease in the AM population (Supplementary Fig. 5b, d). The non-AM population was also found to show higher expression of pro-inflammatory cytokines (Supplementary Fig. 5e).

Differential expression analysis of 4 dpi and −5 dpi BAL macrophages/monocytes showed that CHIT1, MARCO and MRC1 were among the top-ranking genes exhibiting downregulation in the BAL, while genes such as ADAMDEC1^37^ and S100A8^38^ that are associated with monocyte-derived macrophages were among the most upregulated (Supplementary Data 4). These data demonstrate that our observation of an influx of infiltrating macrophages into the BAL at 4 dpi was consistent across multiple definitions of this phenotype.

### Infiltrating macrophages produce the majority of lower airway inflammatory cytokines during acute SARS-CoV-2 infection

Given our observation of the dynamics of pulmonary macrophages within the alveolar space during early SARS-CoV-2 infection, we characterized the transcriptional changes in each macrophage/monocyte population (Fig. 3), and also in conventional myeloid dendritic cells. Myeloid DCs were present at very low frequencies (<2%), and not more than 70 total cells were detected to be harboring mRNA from TNF, IL6 or IL10 after infection. IL1B expression was slightly higher, but accounted for <2.5% of IL1B expressing cells in the BAL at 4 dpi (Supplementary Fig. 6a–e). Several chemokines (CCL4L1, CCL3, CXCL3, CCL2), multiple ISGs, NFKB1A, S100A8, and GZMB were among the most upregulated genes at 4 dpi in BAL populations (Supplementary Data 4). Elevated expression of multiple inflammatory genes, including IL6, TNF, IL10, and IL1B, were observed in the CD163+MRC1+TREM2 Mac and CD163+MRC1− subsets in both Cohort 1 and 2 (Fig. 3i–l and Supplementary Fig. 7) after infection. The infiltrating macrophages were also observed to upregulate multiple chemokines, including those specific for recruiting neutrophils (CXCL3, CXCL8), macrophages (CCL2, CCL3, CCL5, CCL4L1), and activated T cells (CXCL10) as well as multiple ISGs (Supplementary Figs. 7 and 8). When we examined CD163+MRC1+ macrophages, many of the same inflammatory cytokines and gene sets seen in the infiltrating macrophages were elevated at 4 dpi, albeit at much lower magnitude (Supplementary Figs. 7 and 8). Having observed a significantly higher average expression of inflammatory cytokines in infiltrating macrophages compared to CD163+MRC1+ macrophages, we compared the fractions of sequencing reads detected from each of the subsets to assess the overall contribution to inflammatory cytokine production (Fig. 3i). In Cohort 1, at 4 dpi, we observed that the CD163+MRC1+TREM2+ macrophages accounted for 55% of IL6, 57% of TNF, and 86% of IL10 expression while the CD163+MRC1− macrophages accounted for 20% of IL6, 21% of TNF, and 6% of IL10 expression (Fig. 3i). In Cohort 2, we also observed that the infiltrating macrophages contributed more to the expression of most inflammatory cytokines than alveolar macrophages: the expression in CD163+MRC1+TREM2+ and CD163+MRC1− cells was 39.2% and 47% for IL10, 17.4% and 74.9% for IL6, and 22.4% and 46.6% for TNF, respectively (Fig. 3j). To account for potential bias in cell counts in our pooled data, we also examined the contributions of cytokines to the BAL expression in individual animals (Fig. 3k, l). In Cohort 1, the overall trend of higher contribution to inflammatory expression in the infiltrating macrophage populations seen in the pooled data was only observed for IL10 and CCL4L1, largely due to imbalances in cell counts and low statistical power (Fig. 3k). However, in Cohort 2, we observed consistently elevated levels in the infiltrating CD163+MRC1+TREM2+ and CD163+MRC1− macrophage populations (Fig. 3l). For IL10, the mean ± sd expression in CD163+MRC1+ was 17.3 ± 12.4%, compared to 40.4 ± 12.1% in CD163+MRC1+TREM2+ cells (*p* = 0.03) and 42.3 ± 21.2% for CD163+MRC1− (ns) (Fig. 3l). For IL6, the mean ± sd expression in CD163+MRC1+ was 9.4 ± 12.2%, compared to 12.2 ± 24.5% in CD163+MRC1+TREM2+ cells (ns) and 78.4 ± 28.8% for CD163+MRC1− (*p* = 0.065). Additionally, while our observation for IL6 trended to significance, we found a wide variability in the percentages in CD163+MRC1− cells; to address this, we analyzed another set of data^26^ in which we obtained scRNA-Seq expression of BAL macrophage after 2 dpi of SARS-CoV-2 infection—these data also trended to much higher expression of IL6 in the CD163+MRC1− cells (67.1 ± 32.4%) compared to CD163+MRC1+ macrophages (27.7 ± 29.5%)(Supplementary Fig. 9). For CCL4L1, CD163+MRC1+ contributed 9.6 ± 6.3% of expression, in comparison to 23.7 ± 13.5% in CD163+MRC1+TREM2+ cells (*p* = 0.03) and 66.7 ± 18.2% in CD163+MRC1− cells (*p* = 0.03). The contribution of different subsets towards CXCL10 expression was 19.2 ± 16.7% from CD163+MRC1+ compared to 15.3 ± 9.3% from the CD163+MRC1+TREM2+ and 65.4 ± 15.2% from the CD163+MRC1− populations. Overall, these data indicate that the infiltrating macrophage populations are responsible for the majority of lower airway inflammatory cytokine production during acute SARS-CoV-2 infection.

To validate the increase in infiltrating myeloid populations, we quantitated by flow cytometry the frequency of CCR2+ myeloid populations in peripheral blood and BAL from an additional six SARS-CoV-2-infected rhesus macaques (Supplementary Fig. 10). CCR2 has been demonstrated to regulate monocyte infiltration into the lung parenchyma of SARS-CoV-2 infected mice^39^ and its expression is upregulated in the BAL of infiltrating macrophages in NHPs infected with influenza virus^35^. Consistent with our observation of elevated infiltrating myeloid cells by scRNA-Seq, we found that there was a concomitant increase in the frequency of CCR2+ CD14−CD16+ and CD14+CD16+ monocytes at 2 dpi. These results further support the infiltration of inflammatory monocytes in BAL after SARS-CoV-2 infection.

### Identification of pro-inflammatory subsets in human SARS-CoV-2 infection corresponding to NHP immunophenotypes

**Fig. 4:**
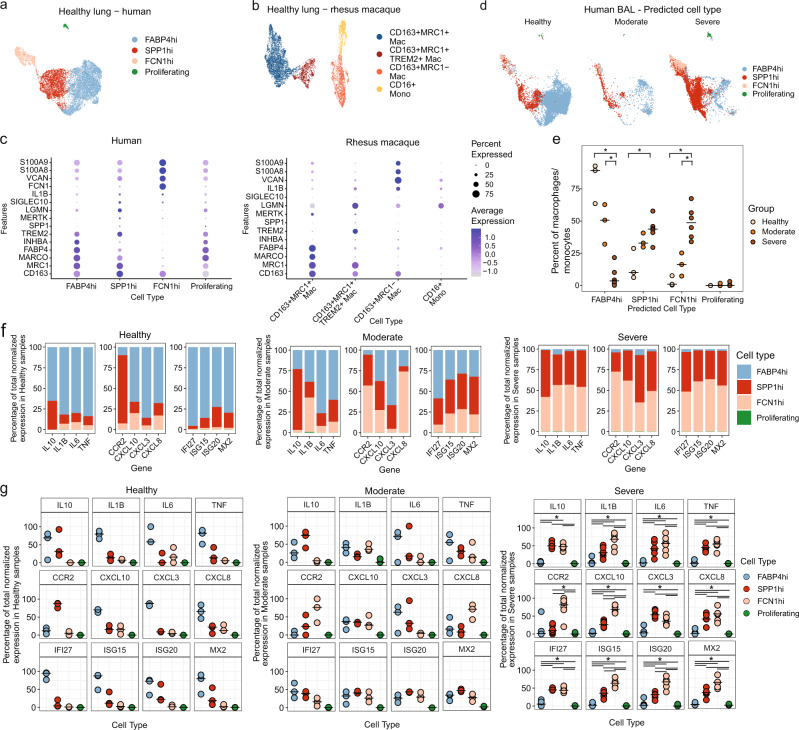
Comparison of rhesus and human macrophage subsets. **a** UMAP of macrophage subsets in lungs from six healthy human donors (GEO: GSE135893). **b** UMAP of macrophage subsets in lungs from three healthy rhesus macaques (GEO: GSE149758). **c** DotPlots showing the expression of marker genes. The color gradient represents the level of expression and the size of the dot represents the percentage of cells expressing a given gene. **d** UMAP of BAL samples from human donors that are healthy (*n* = 3), or suffering from moderate (*n* = 3) or severe (*n* = 6) COVID-19 (GEO: GSE145926) mapped to the healthy lung reference using the Seurat MapQuery function. **e** Percent of predicted cell types out of all macrophage/monocytes in each human BAL sample. The black bar indicates the median. Statistical analysis was performed using pairwise two-tailed Mann–Whitney *U* test in R v4.2.2. *p* value * = 0.02. Contribution of each predicted macrophage/monocyte subsets in human BAL toward the production of the pro-inflammatory genes and ISG—pooled (**f**) and individual (**g**). The percentage contribution was calculated by dividing the sum of normalized expression of a given gene in a macrophage/monocyte subset by the sum of the normalized expression of the gene in all macrophage/monocyte subsets. The black bars represent the median. Statistical analysis was performed using two-tailed Wilcoxon signed rank test in R v4.2.2. *p value 0.03 for all except FABP4 vs. Proliferating for IL6, IL1B, TNF, CXCL3, CXCL8 and SPP1hi vs. Proliferating for CCR2: *p* value = 0.04. Source data (**e**–**g**) are provided as a Source Data file.

To translate our findings in the NHP model to human SARS-CoV-2 infection, we used a similar bioinformatic approach to that employed to define rhesus myeloid. We used macrophages/monocytes from publically available scRNA-Seq dataset of lungs from six healthy human donors (GEO: GSE135893^40^) and classified these based on a recent classification into FABP4hi, SPP1hi, FCN1hi and proliferating macrophages^41^ (Fig. 4a). When the canonical marker genes were compared between the healthy lung macrophages/monocytes of human and rhesus macaque, we found that there were comparable populations between the two (Fig. 4a–c). Namely the CD163+MRC1+ rhesus subset was highly similar to the FABP4hi human subset; the CD163+MRC1+TREM2+ rhesus subset was congruent with the SPP1hi human subset; and the CD163+MRC1− macrophages and CD16+ monocytes rhesus were transcriptionally similar to the FCN1hi human subset. Next, we combined data from all macrophage/monocyte cells from the six healthy human samples with the three healthy rhesus samples and applied reference-based integration in Seurat using the human samples as reference (Supplementary Fig. 11a, b). We looked at the distribution of different human and rhesus cell types in each cluster and found that the earlier observations regarding the similarity of subsets based on canonical markers was further supported by the global gene expression of these cells (Supplementary Fig. 11c). Finally, to test the robustness of our cellular classifications to identify macrophage subsets between species accurately, we generated gene signatures for each subset/species combination and tested for enrichment in the opposite species; for all comparisons, a signature scored highest with its corresponding opposite subset (Supplementary Fig. 11d).

Using the healthy human dataset as reference, we classified macrophages/monocytes from the myeloid cluster (Supplementary Fig. 11e, f) in a publically available scRNA-Seq dataset of human BAL samples from healthy donors or subjects with moderate or severe COVID-19 infection^8^ (Fig. 4d and Supplementary Fig. 11g). As reported in the original study^8^, we found that there was a significant increase in the SPP1hi and FCN1hi subsets with COVID-19 infection while the FABP4hi population that is largely representative of the resident alveolar macrophages was found to be significantly reduced in both moderate and severe COVID-19 infection (Fig. 4d, e). In addition, we also looked at the contribution of these different populations toward inflammation and found that the non-resident SPP1hi and FCN1hi subsets were largely responsible for the expression of pro-inflammatory mediators in patients with severe COVID-19 (Fig. 4f and Supplementary Fig. 12). Respectively, (FABP4hi, SPP1hi, and FCN1hi) the contribution of each population to overall expression was: IL10 (1.3%, 56.7%, 42%), IL1B (6.6%, 37%, 56%), IL6 (2.4%, 40.8%, 56.7%), TNF (1.6%, 44.1%, 54.1%), CXCL10 (1.8%, 36.5%, 61.7%) and CXCL8 (2.6%, 48.1%, 49.1%) (Fig. 4f). Of note, ISG expression (IFI27, ISG15, ISG20, MX2) was significantly upregulated in the SPP1hi, and FCN1hi populations relative to the FABP4hi in severe disease but not in moderate cases. Lastly, when we examined the contribution of cytokine expression in by individual patients in these dataset, we noted that for severe COVID-19, we observed that the infiltrating populations had significantly higher expression of IL10 (mean ± sd SPP1hi 50.9 ± 8.7%, *p* = 0.03; FCN1hi 46.9 ± 8.3%, *p* = 0.03), IL1B (mean ± sd SPP1hi 31.7 ± 14.9%, *p* = 0.03; FCN1hi 63.3 ± 20.1%, *p* = 0.03), TNF (mean ± sd SPP1hi 43.4 ± 9.3%, *p* = 0.03; FCN1hi 53.7 ± 13.7%, *p* = 0.03), CXCL10 (mean ± sd SPP1hi 27.3 ± 9.5%, *p* = 0.03; FCN1hi 69.8 ± 11.1%, *p* = 0.03), CXCL3 (mean ± sd SPP1hi 55.9 ± 9.6%, *p* = 0.03; FCN1hi 37.6 ± 13%, *p* = 0.06) and CXCL8 (mean ± sd SPP1hi 42.7 ± 14%, *p* = 0.03; FCN1hi 53.4 ± 17.4%, *p* = 0.03) compared to the FABP4hi population (mean ± sd for IL10 2.2 ± 2.8%, IL1B 4.7 ± 8.2%, TNF 2.7 ± 4.5%, CXCL10 2.6 ± 2.9%, CXCL3 6.4 ± 9.5% and CXCL8 3.8 ± 5.9%) (Fig. 4g). Collectively, these analyses demonstrate that the myeloid subsets defined transcriptionally in RMs have analogous populations in the human lung, and an overall concordance in their expansion and contribution to SARS-CoV-2 induced inflammation in the lower airway.

### Baricitinib treatment prevents the influx of inflammatory IM into the lower airway

**Fig. 5:**
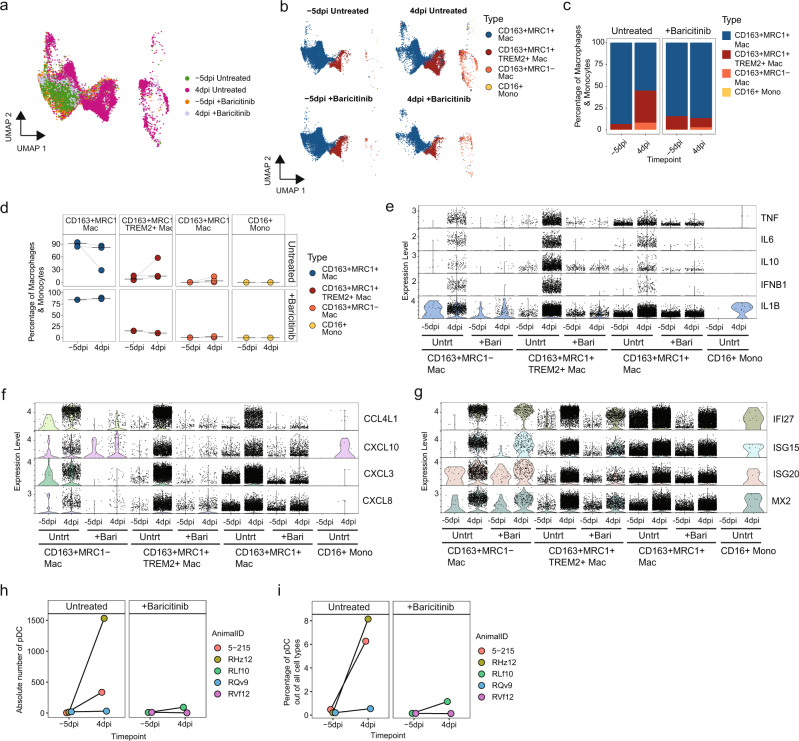
Baricitinib reduced the influx of pro-inflammatory macrophages in addition to the pro-inflammatory gene expression profile. **a** Projection of macrophages/monocytes from −5 dpi and 4 dpi 10X BAL samples from three untreated and two baricitinib-treated rhesus macaques on the reference UMAP of uninfected lung macrophages/monocytes (NCBI GEO: GSE149758). **b** UMAP split by treatment and timepoint showing predicted cell annotations based on mapping to the reference lung macrophages/monocytes. Percentage of a given macrophage/monocyte subset of all the macrophages/monocytes in the BAL samples—pooled (**c**) and individual (**d**). Violin plots showing expression of pro-inflammatory cytokines (**e**), chemokines (**f**) and ISG (**g**) in the different macrophage/monocyte subsets in BAL 10X samples from baricitinib-treated and untreated samples. **h** Absolute number of pDC in BAL samples from scRNA-Seq and **i** Percentage of pDC out of all cells in the BAL samples from scRNA-Seq. Source data (**c**, **d**, **h**, **i**) are provided as a Source Data file.

Baricitinib is a JAK1/2 inhibitor approved for the treatment of active rheumatoid arthritis that was recently approved by FDA for COVID-19 treatment in certain hospitalized adults^42^, and reported to reduce mortality when administered as monotherapy^43^ or in combination with remdesivir^25^. In our earlier study, using data from the Cohort 1 and baricitinib-treated animals (Fig. 1a), we found that baricitinib was able to suppress the expression of pro-inflammatory cytokines in BAL of RMs infected with SARS-CoV-2^20^. Here, we extended this study to further characterize the impact of baricitinib on the myeloid populations in the airway from five RMs before infection (−5 dpi) and at 4 dpi, with three RMs that remained untreated and two that received baricitinib. We found that 2 days of baricitinib administration virtually abrogated the influx of infiltrating macrophages into the alveolar space at 4 dpi, as we did not detect any increase in the CD163+MRC1− or CD163+MRC1+TREM2+ populations in baricitinib-treated animals (Fig. 5a–d). This observation was consistent using classifications of macrophages either based on mapping to 10X lung reference or using bulk sorted AM/IM cells (Fig. 5a–d and Supplementary Fig. 5a–d). In addition to preventing the influx of infiltrating macrophages, baricitinib treatment also resulted in significantly lower expression of inflammatory cytokines and chemokines, but the ISG expression remained comparable to untreated animals (Fig. 5e–g and Supplementary Fig. 5e). In summary, these data further elucidate the mechanism of action by which baricitinib treatment abrogates airway inflammation in SARS-CoV-2 infection^20^, by demonstrating its ability to block infiltration of discrete pro-inflammatory macrophage populations into the alveolar compartment. Similar to our observations using flow cytometry, there was an increase in the abundance of pDC at 4 dpi in the BAL detected by scRNA-Seq data, however this increase was abrogated in baricitinib-treated animals (Fig. 5h, i).

## Discussion

The mechanisms by which SARS-CoV-2 infection establishes severe disease remain largely unknown, but remain a key priority for reducing the toll of the COVID-19 pandemic. As the appearance of symptoms range from 2–14 days after SARS-CoV-2 infection, characterization of the early immunological events using clinical samples is challenging. Here, we utilized the RM model of SARS-CoV-2 infection and an integrated systems analysis to dissect the immune response during hyperacute infection. Our findings were: (1) SARS-CoV-2 infection initiated a robust Type I IFN response in the blood and lower airway apparent at 1–2 dpi; (2) SARS-CoV-2 induced a rapid influx of two infiltrating macrophage populations, into the bronchoalveolar space, which produced the majority of inflammatory cytokine production; and (3) the mechanism of action of baricitinib, a drug recently authorized by FDA for use in the treatment of COVID-19 for certain hospitalized adults^42^, is to abrogate infiltration of these inflammatory cells into the airway. Our data present, to date, the most comprehensive analysis of the immunopathological events occurring during hyperacute SARS-CoV-2 infection.

Using our reference datasets of RM lung macrophages, we identified two myeloid cell subsets, both clearly distinct from alveolar macrophages, infiltrating the airway after SARS-CoV-2 infection, that were the main producers of lower airway inflammatory cytokines and chemokines. One population, defined as CD163+MRC1+TREM2+ cells, were highly similar to murine definitions of infiltrating CCR2+ monocytes. The second, CD163+MRC1−, largely resembled interstitial macrophages. Our data are consistent with a recent observation of a rapid (3 dpi) increase of IMs in the BAL of RMs using flow cytometry^21^. Similarly, an accumulation of non-AMs (defined as CD16+CD206-HLA-DR+/CD11b+), and reciprocal reduction of AMs, has been observed in the BAL and lungs of infected RMs and AGMs^23^. We found that these myeloid subsets, defined transcriptionally in NHPs, had analogous populations in the human lung. Lastly, our data are consistent with our recent findings in the murine model, in which SARS-CoV-2 elicited recruitment of circulating monocytes to the lung parenchyma, but was significantly abrogated in CCR2-deficient mice^39^. The CD163+MRC1+Mac/AM-like subset also contributed to the inflammatory milieu, producing IL6, TNF, and IL10, albeit in significantly lower quantities.

It is important to note that our observations were during hyperacute infection, and that our animals did not develop severe disease, so although our data indicate that these infiltrating populations orchestrate early inflammation and may contribute to airway pathogenesis, we cannot formally make this link. However, this model is consistent with recent data by Ren et al.^44^, who observed a significant loss of MARCO expression in BAL-resident myeloid populations of patients with severe COVID-19 relative to those with moderate disease, similar to our observations, in which the appearance of infiltrating macrophages diluted the population of MARCO+ macrophages. Those observations, taken together with our data, suggest that the inflammatory macrophage phenotype we identify here may be preferentially retained in the lower airway of patients with severe COVID-19. Additionally, we demonstrated that in vivo treatment with the JAK1/2 inhibitor baricitinib, which has demonstrated efficacy in reducing severe disease, was able to virtually abrogate the recruitment of these inflammatory macrophages into the airway, providing an additional mechanistic link with the development of COVID-19-related pathogenesis.

In addition to inflammatory cytokines, we observed that the infiltrating macrophage subsets produced high levels of IL10, and were enriched in IL10 signaling pathways. Lung IM’s are considered to be a “professional IL10-producing cell” producing IL10 at both a steady state and in response to innate stimuli (LPS, unmethylated CpGs)^45^. The majority of data to date has demonstrated an immunoregulatory, protective role for IMs in murine models of asthma, lung fibrosis, and allergen induced inflammation^30^. However, while the pro-inflammatory potential of IMs has been relatively understudied, they have been demonstrated to be efficient at producing IL6 and TNF in response to TLR ligands^46^. Given our observations of high IL10 production in infiltrating macrophages, we cannot exclude a potential immunoregulatory role for this subset, and indeed, it presents an interesting hypothesis in which the balance of infiltrating IM vs. TREM2+ macrophages into the bronchoalveolar space determines the pathogenic outcome of SARS-CoV-2 infection. Lastly, recent publications have reported that lung IMs may be comprised of two, or even three, functionally distinct populations, defined by an axis of expression of Lyve1, MHC, CD169, and CD11c^29–32^. We did not observe separate clustering amongst BAL IMs, nor differential expression amongst these markers, and further work is needed to understand the congruency of macaque macrophage subsets with those identified in the murine model.

We observed a very rapid and robust induction of the Type I IFN pathway at 1–2 dpi, characterized by elevated pDCs in the airway and blood, IFNA and IFNB transcripts and protein, upregulated ISGs, and increased granzyme B in NK cells. The Type I IFN response in SARS-CoV-2 infection has been intensely studied: in vitro infection of airway epithelial cells have consistently resulted in a muted ISG response^47^, and patients developing severe COVID-19 have been reported to have higher incidence of mutations in IFN response genes, or elevated levels of autoantibodies against IFN-response genes (reviewed in refs. ^6,48–55^). Our data, in which the IFN response peaked at 2 dpi and had largely abated by 10/11 dpi, provides well defined kinetics of the ISG response, and similar observations have been reported in other NHP studies^21,22^. The rapid and short-lived nature of the IFN response underscores the difficulty in interpreting the IFN response in clinical samples.

Our multi-parametric analyses demonstrated an increase of pDCs at 2 dpi that coincided with the peak of ISG production, IFNA/B detection, and NK cell activation, thus implicating pDCs as the primary cell orchestrating the IFN response in the lower airway. We had previously observed a reduction in peripheral blood pDCs frequencies and activity in human SARS-CoV-2 infection^56^, and other have reported signatures of pDCs apoptosis that predicted lower IFN-I responses^57^. Taken in the context of these clinical findings, our observation of pDC accumulation in the BAL indicates that they undergo rapid mobilization from the blood to the lower airway, and this suggests they likely drive early protective innate immune responses. However, pDCs may also contribute to pathological inflammation; and future interventional studies targeting the pDC/IFN axis in animal models will be necessary to test these hypotheses.

In our prior study, we demonstrated the ability of baricitinib to block airway pro-inflammatory cytokine production in SARS-CoV-2-infected RMs while preserving Type I IFN responses^20^. The efficacy of baricitinib to treat severe COVID-19 was recently tested in two Phase 3 clinical trials (ACTT-2 and COV-BARRIER) and recently received approval as monotherapy to treat COVID-19 in patients requiring supplemental oxygen or mechanical ventilation^42^. To date, nearly 1 M patients have received baricitinib to treat severe COVID-19 disease, underscoring the importance for understanding its mechanism of action. Here, we extended our original findings to demonstrate that baricitinib blocked the influx of inflammatory macrophages into the bronchoalveolar space. These data add to our mechanistic understanding of the action of baricitinib, and provide a potential explanation for the disparity of baricitinib’s impact on IFN vs. IL6/TNF signaling when considering the timing of the drug administration. We administered baricitinib at 2 dpi, after the peak influx of pDCs, but before the likely appearance of the inflammatory macrophages at 3-4 dpi. The ongoing ISG response, and suppressed TNF/IL6 response, suggest that the primary mechanism by which baricitinib protects the airway is by blocking recruitment of inflammatory cells to the bronchoalveolar space. Of note, in the presence of baricitinib and concomitant elimination of infiltrating monocytes/macrophages, viral load in the BAL was unchanged compared to controls, suggesting that these populations exert minimal control of virus levels. However, as the rhesus model of SARS-CoV-2 tends to be consistent with mild COVID-19 in the majority of studies, it will be critical to examine the mechanism of baricitinib in a model of severe disease. In regards to guiding future clinical applications of baricitinib, our data suggest that timing is critical, and would favor earlier drug administration. Additionally, given the pervasiveness of SARS-CoV-2, and growing capacity for re-infection, future studies on the application of baricitinib for treatment second infections would be of benefit.

Our study had some limitations; first, while the RM/SARS-CoV-2 model has rapidly been adopted by several groups for pre-clinical testing of anti-COVID drugs and vaccines, no group has demonstrated overt, reproducible symptomatic disease^11^. Thus, linking early immunological events to the development of severe COVID-19 requires validation in human studies, such as the observations of reduced MARCO expression in the airway myeloid populations of severe COVID-19 patients noted above^44^. Additionally, to estimate the overall contribution to inflammatory cytokine production in macrophages, we calculated the fraction of sequencing reads for a given mRNA transcript assigned to a subset, however we did not measure protein quantities on a single-cell level, and instead were limited to assessing overall levels in BALF. Prior studies have attempted to estimate the correlation of cytokine mRNA to secreted protein levels and have reported good agreement for TNF, IL6, CXCL10 and CXCL8, but poorer concordance for IL10 and IL1B^58^. Another drawback was the relatively low power of our study. While our observations at 0, 1, and 2 dpi were *n* = 8, we were limited to *n* = 4 for day 4–10 observations. For our scRNA-Seq experiment, we addressed power issues conducting our analysis on two independent cohorts for a total of nine animals. Overall, there was no lack of statistical power for our key observations.

While the global vaccine rollout has made great strides to reduce the transmission and severity of SARS-CoV-2 infection, millions of people remain vulnerable. Understanding the early events of SARS-CoV-2 infection, and the mechanisms by which clinically approved drugs afford protection, remains a global priority. In this study, we have identified two populations of inflammatory myeloid cells that are responsible for the preponderance of airway inflammation in acute SARS-CoV-2 infection. We also demonstrated that treatment with baricitinib, recommended by the World Health Organization for treatment of severe COVID-19 in January 2022, blocked infiltration of these inflammatory cells into the alveolar space. These data identify both a key druggable target (airway infiltrating macrophages), and an efficacious mechanism by which to lower airway inflammation, and should prove useful for identifying additional drugs to reduce the incidence and mortality of severe COVID-19 disease.

## Methods

### Animal and SARS-CoV-2 infections

The animal care facilities at ENPRC are accredited by the Association for Assessment and Accreditation of Laboratory Animal Care (AAALAC) as well as the U.S. Department of Agriculture (USDA). Emory University’s Institutional Animal Care and Use Committee (IACUC) reviewed and approved all animal experiments under permit PROTO202000035. All procedures were performed as per the institutional regulations and guidelines set forth by the NIH’s Guide for the Care and Use of Laboratory Animals (8th edition) and were conducted under anesthesia and appropriate follow-up pain management to minimize animal suffering. Eight (4 females, 4 males, aged >11 years) specific-pathogen-free Indian-origin rhesus macaques were infected via intranasal and intratracheal routes with 1.1 × 10^6^ plaque-forming units (PFU) SARS-CoV-2 as previously described^20^ and were maintained in the ABSL3 at YNPRC (IACUC permit PROTO202000035). The processing of nasopharyngeal swabs, BAL and mononuclear cells was performed as described previously^20^. Six (2 females, 4 males, aged >6 years) additional specific-pathogen-free Indian-origin rhesus macaques were added to the study (IACUC permit PROTO202100003) and were also infected via intranasal and intratracheal routes with 1.1 × 10^6^ plaque-forming units (PFU) SARS-CoV-2 for characterization of CCR2 expression in whole blood and BAL.

The eight animals under IACUC permit PROTO202000035 were age and sex matched between untreated control and baricitinib-treated experimental arms, with two females and two males assigned to each respective arm. Cohort 2 (IACUC permit PROTO202100003) was comprised of two females and four males, all of which served as untreated controls. Efforts to include equal numbers of females and males in Cohort 2 were made. However, female macaques were limited at the time of Cohort 2 animal assignment due to breeding demands. In total, between Cohort 1 and 2, four female and six male untreated controls were included in this study.

### Viral stocks

The viral stocks used for infecting the 8 RMs on IACUC permit PROTO202000035 were previously described^20^. SARS-CoV-2 (NR-52281: BEI Resources, Manassas, VA; USA-WA1/2020, Lot no. 70033175) was passaged on Vero E6 cell line (African Green Monkey Kidney cell line; CRL-1586, ATCC) at a MOI of 0.01. The TCID_50_ method was used to propagate and titrate SARS-CoV-2 followed by storage of aliquots at −80 °C. The infectious dose delivered was determined by back titration of viral stocks via plaque assay. The virus stock was sequenced to confirm the presence of furin cleavage motif. The viral stocks used had less than 6% of genomes with a mutation that may abrogate furin cleavage. The 6 RMs on IACUC permit PROTO202100003 were infected with the viral stock NR-53899: BEI Resources, Manassas, VA; USA-WA1/2020.

### Determination of viral load RNA

The SARS-CoV-2 genomic and sub-genomic RNA was quantified in nasopharyngeal swabs, throat swabs, and BAL as previously described^18,20^. The swabs were kept in 1 ml of Viral Transport Medium (VTM-1L, Labscoop, LLC). The viral RNA was extracted from fresh specimens of nasopharyngeal (NP) swabs, throat swabs, and BAL manually using the QiaAmp Viral RNA mini kit as per the manufacturer’s protocol. For genomic RNA, CDC designed N2 primer and probe set: CoV2-N2-F: 5’-TTACAAACATTGGCCGCAAA-3’, CoV2-N2-R: 5’-GCGCGACATTCCGAAGAA-3’, and CoV2- N2-Pr: 5’-FAM-ACAATTTGCCCCCAGCGCTTCAG-BHQ-3’^59^ were used for quantitative PCR (qPCR). For sub-genomic RNA, the primer and probe sequences for E gene sub-genomic mRNA transcript^60^ were used: SGMRNA-E-F: 5’-CGATCTCTTGTAGATCTGTTCTC-3’, SGMRNA-E-R: 5’-ATATTGCAGCAGTACGCACACA-3’, and SGMRNA-E-Pr: 5’-FAM-ACACTAGCCATCCTTACTGCGCTTCG-3’. The qPCR reactions were performed with the TaqMan Fast Virus 1-step Master Mix using the manufacturer’s cycling conditions, 200 nM of each primer, and 125 nM of the probe in duplicate. In total, 257 copies/ml VTM/plasma/BAL was the limit of detection for this assay. The CDC RNase P p30 subunit qPCR, modified for rhesus macaque specific polymorphisms, was used to verify sample quality using the following primer and probe sequences: RM-RPP30-F 5’-AGACTTGGACGTGCGAGCG-3’, RM-RPP30-R 5’-GAGCCGCTGTCTCCACAAGT-3’, and RPP30-Pr 5’-FAM-TTCTGACCTGAAGGCTCTGCGCG-BHQ1-3’. The RNA integrity and sample quality was verified by running a single well from each extraction.

### Tissue processing

NP swabs were collected under anesthesia by using a clean rayon-tipped swab (Thermo Fischer Scientific, BactiSwab NPG, R12300) placed ~2–3 cm into the nares. Oropharyngeal swabs were collected under anesthesia using polyester tipped swabs (Puritan Standard Polyester Tipped applicator, polystyrene handle, 25-806 2PD, VWR International) to streak the tonsils and back of throat bilaterally (throat/pharyngeal). The swabs were dipped in 1 ml viral transport media (Viral transport Media, VTM-1L, Labscoop, LLC) and vortexed for 30 s, and the eluate was collected.

To collect BAL, a fiberoptic bronchoscope (Olympus BF-XP190 EVIS EXERA III ULTRA SLM BRNCH and BF-P190 EVIS EXERA 4.1 mm) was manipulated into the trachea, directed into the primary bronchus, and secured into a distal subsegmental bronchus upon which 35–50 ml of normal saline (0.9% NaCl) was administered into the bronchus and re-aspirated to obtain a minimum of 20 ml of lavage fluid. BAL was filtered through a 70 μm cell strainer and multiple aliquots were collected for viral loads. Next, the remaining BAL was centrifuged at 2200 rpm for 5 min and the BAL fluid supernatant was collected for mesoscale analysis. Pelleted BAL cells were resuspended in R10 and used for downstream analyses.

Mononuclear cells were counted for viability using a Countess II Automated Cell Counter (Thermo Fisher) with trypan blue stain and were cryo-preserved in aliquots of up to 2 × 10^7^ cells in 10% DMSO in heat-inactivated FBS. Whole tissue segments (0.5 cm^3^) were snap frozen dry, or stored in RNAlater (Qiagen), or Nuclisens lysis buffer (Biomerieux) for analyses of compound distribution, RNA-seq, and tissue viral quantification, respectively.

### Immunophenotyping

In total, 23-parameter flow cytometric analysis was performed on fresh EDTA whole blood^61^ and BAL mononuclear cells from SARS-CoV-2 infected RMs as described previously^20^ using anti-human monoclonal antibodies (mAbs), which we and others^20,62,63^, including databases maintained by the NHP Reagent Resource (MassBiologics), have shown as being cross-reactive in RMs.

A panel of the following mAbs was used for the longitudinal phenotyping of innate immune cells in whole blood (500 μl), and mononuclear cells (10^6^ cells) derived from BAL from Cohort 1 and baricitinib-treated animals: anti-CD20-BB700 (clone 2H7; 2.5 ul; cat. # 745889), anti-Ki-67-BV480 (clone B56; 5 ul; cat. # 566109), anti-CD14-BV605 (clone M5E2; 2.5 ul; cat. # 564054), anti-CD56-BV711 (clone B159; 2.5 ul; cat. # 740781), anti-CD115-BV750 (clone 9-4D2-1E4; 2.5 ul; cat. # 747093), anti-CD3-BUV395 (clone SP34-2; 2.5 ul; cat. # 564117), anti-CD8-BUV496 (clone RPA-T8; 2.5 ul; cat. # 612942), anti-CD45-BUV563 (clone D058-1283; 2.5 ul; cat. # 741414), anti-CCR2-BUV661 (clone LS132.1D9; 2.5 ul; cat. # 750472), anti-CD16-BUV737 (clone 3G8; 2.5 ul; cat. # 564434), anti-CD69-BUV805 (clone FN50; 5 ul; cat. # 748763), and Fixable Viability Stain 700 (2 ul; cat. # 564997) all from BD Biosciences; anti-CD38-FITC (clone AT1; 5 ul; cat. # 60131FI) from STEMCELL Technologies; anti-CD161-BV421 (clone HP-3G10; 5 ul; cat. # 339914), anti-HLA-DR-BV650 (clone L243; 5 ul; cat. # 307650), anti-CD11c-BV785 (clone 3.9; 5 ul; cat. # 301644), anti-CD11b-PE (clone ICRF44; 2.5 ul; cat. # 301306), and anti-CD123-APC-Fire750 (clone 315; 2.5 ul; cat. # 306042) all from Biolegend; anti-GranzymeB-PE-TexasRed (clone GB11; 2.5 ul; cat. # GRB17) from Thermo Fisher; anti-CD66abce-PE-Vio770 (clone TET2; 1 ul; cat. # 130-119-849) from Miltenyi Biotec; and anti-CD27-PE-Cy5 (clone 1A4CD27; 2.5 ul; cat. # 6607107) and anti-NKG2A-APC (clone Z199; 5 ul; cat. # A60797) from Beckman Coulter (Supplementary Fig. 4).

For Cohort 2 animals, a different panel of the following mAbs was used for the longitudinal phenotyping of innate immune cells in whole blood (500 μl), as described in ref. ^26^, and mononuclear cells (2 × 10^6^ cells) derived from BAL: anti-CD20-BB700 (clone 2H7; 2.5 μl; cat. # 745889), anti-CD11b-BV421 (clone ICRFF44; 2.5 μl; cat. # 562632), anti-Ki-67-BV480 (clone B56; 5 μl; cat. # 566109), anti-CD14-BV605 (clone M5E2; 2.5 μl; cat. # 564054), anti-CD56-BV711 (clone B159; 2.5 μl; cat. # 740781), anti-CD163-BV750 (clone GHI/61; 2.5 μl; cat. # 747185), anti-CD3-BUV395 (clone SP34-2; 2.5 μl; cat. # 564117), anti-CD8-BUV496 (clone RPA-T8; 2.5 μl; cat. # 612942), anti-CD45-BUV563 (clone D058-1283; 2.5 μl; cat. # 741414), anti-CCR2-BUV661 (clone LS132.1D9; 2.5 μl; cat. # 750472), anti-CD16-BUV737 (clone 3G8; 2.5 μl; cat. # 564434), anti-CD101-BUV805 (clone V7.1; 2.5 μl; cat. # 749163), anti-CD169-PE (clone 7-239; 2.5 μl; cat. # 565248), and anti-CD206-PE-Cy5 (clone 19.2; 20 μl; cat. # 551136) and Fixable Viability Stain 700 (2 μl; cat. # 564997) all from BD Biosciences; anti-ACE2-AF488 (clone Polyclonal; 5 μl; cat. # FAB9332G-100UG) from R&D; anti-HLA-DR-BV650 (clone L243; 5 μl; cat. # 307650), anti-CD11c-BV785 (clone 3.9; 5 μl; cat. # 301644), and anti-CD123-APC-Fire750 (clone 315; 2.5 μl; cat. # 306042) all from Biolegend; anti-GranzymeB-PE-TexasRed (clone GB11; 2.5 μl; cat. # GRB17) from Thermo Fisher; anti-CD66abce-PE-Vio770 (clone TET2; 1 μl; cat. # 130-119-849) from Miltenyi Biotec; anti-NKG2A-APC (clone Z199; 5 μl; cat. # A60797) from Beckman Coulter. mAbs for chemokine receptors (i.e., CCR2) were incubated at 37 C° for 15 min, and cells were fixed and permeabilized at room temperature for 15 min with Fixation/Permeabilization Solution Kit (BD Biosciences; cat. #554714). For each sample, a minimum of 1.2 × 10^5^ stopping gate events (live CD3+ T-cells) were recorded. All samples were fixed with 4% paraformaldehyde and acquired within 24 h of fixation. Acquisition of data was performed on a FACSymphony A5 (BD Biosciences) driven by FACS DiVa Version 8.0 software and analyzed with FlowJo (version 10.7; Becton, Dickinson, and Company). The gating strategy is shown in Supplementary Fig. 1.

### Bulk RNA-Seq library and sequencing

The data for −5 dpi, 2 dpi and 4 dpi for bulk BAL samples was obtained from our previous study^20^. Here we expanded our study to include 7 dpi and 10 dpi/11 dpi samples for BAL and −5 dpi, 1 dpi, 2 dpi, 4 dpi, 6 dpi, 7 dpi, 8 dpi and 10/11 dpi for PBMC. Cell suspensions were prepared in BSL3, for bulk RNA-Seq, 250,000 cells (PBMCs) or 100,000 cells (BAL) were lysed directly into 700 ul of QIAzol reagent. The RNeasy Mini or Micro kits (QIAGEN) with on-column DNase digestion was used to isolate RNA. The quality of RNA was determined using an Agilent Bioanalyzer and the cDNA synthesis was carried out using the total RNA with Clontech SMARTSeq v4 Ultra Low Input RNA kit (Takara Bio) as per the manufacturer’s instructions. Dual-indexed bar codes were appended to the amplified cDNA after fragmenting using the NexteraXT DNA Library Preparation kit (Illumina). Agilent 4200 TapeStation was used to validate the libraries by capillary electrophoresis and the libraries were pooled at equimolar concentrations. The libraries were sequenced on an Illumina NovaSeq6000 at 100SR, yielding 20–25 million reads per sample.

### Bulk RNA-Seq analysis

The BCL files were converted to Fastq using bcl2fastq v2.20.0.422. The genome sequences for Macaca mulatta (Mmul10 Ensembl release 100), SARS-CoV-2 (strain MN985325.1—NCBI) and ERCC sequences were combined to build a STAR index (v2.7.3a) as described previously and the reads were aligned to this reference^20^. The ReadsPerGene files were converted into the htseq format and were then imported in DESeq2 v1.24.0^64^ using the DESeqDataSetFromHTSeqCount function. The design used was: ∼ Subject + Group * Timepoint where Group distinguished between samples that were untreated or treated with baricitinib during the time course. Differentially expressed genes for BAL and PBMC were determined using a threshold of *p*adj <0.05, fold-change >2 and filtering out lowly expressed genes where all of the samples at a particular timepoint were required to have detectable expression by normalized reads >0 for that gene.

The input for GSEA v4.1.0^65^ was the regularized log expression values obtained from DESeq2. The following gene sets were used for GSEA analysis: Hallmark and Canonical pathways (MsigDB), NHP ISGs^66^ and Rheumatoid arthritis (KEGG map05323). Since the gene names are largely consistent between the rhesus monkey and human genome references, they were used unaltered with human MsigDB gene sets. The default parameters were used to run GSEA with gene_set permutation type. Volcano plots of differential expression at each timepoint were generated with EnhancedVolcano (v1.8.0) R library^67^. The regularized log expression values from DESeq2 were used to generate heatmaps using the ComplexHeatmap (v2.0.0) R library^68^.

### scRNA-Seq analysis

The filtered count matrices for BAL were obtained from GEO GSE159214^20^. For each BAL sample from SARS-CoV-2 infected rhesus macaque, the count matrix was filtered to include only the protein coding genes. Genes encoded on Y chromosome, mitochondrial genes, RPS and RPL genes, B-cell receptor and T-cell receptor genes, and HBB were filtered out. The Seurat library v4.0.4^34^ was used to perform the analysis. The following parameters were used to filter cells: (1) nFeature_RNA ≥ 200 and ≤4000, (2) % of HBB gene <10, (3) % of mitochondrial genes <20, (4) % of RPS/RPL genes <30 and (5) log10(nFeature_RNA) / log10(nCount_RNA) ≥ 0.8. The number of cells from each sample that passed QC metrics are included in Supplementary Data 5. All the BAL samples from each animal at −5 dpi and 4 dpi were then integrated as per the Seurat integration pipeline^69^ using the default CCA method after normalizing the samples using SCTransform method. The first 30 dimensions were used with RunUMAP and FindNeighbors functions.

For identification of DC, all the BAL samples from −5 dpi and 4 dpi for both treated and untreated rhesus macaques were integrated using CCA. The clusters were determined using the FindClusters function in Seurat and the cells were annotated using SingleR (v1.4.0) (Supplementary Fig. 10a, b). The seurat cluster 11 was classified as pDC based on the expression of canonical markers (Supplementary Fig. 10c). The clusters 17 and 22 were classified as mDC and activated mDC based on expression of marker genes reported previously^70^.

For getting the subset of macrophages/monocytes, the largest cluster primarily comprised of macrophages/monocytes annotated by SingleR (BluePrintEncode database) was selected. Cells that were annotated as another cell type in this cluster were filtered out. The macrophages/monocytes from all BAL samples were then split into individual samples, normalized using SCTransform method and then integrated again using 30 dimensions. We used the FindMarkers function in Seurat to test for differential expression using the MAST (v1.16.0) method^71^.

The macrophages/monocytes from BAL samples were annotated into subsets using two approaches—(1) mapping to macrophages/monocytes from lung reference using Seurat and (2) using bulk sorted cells as reference with SingleR^28^. The 10X lung scRNA-seq data from three uninfected macaque was obtained from a published study (NCBI GEO: GSE149758)^33^. The following parameters were used to filter cells: (1) nFeature_RNA ≥ 200 and ≤4000, (2)% of mitochondrial genes <20, (3)% of RPS/RPL genes <50 and (4) log10(nFeature_RNA) / log10(nCount_RNA) ≥ 0.8. The samples were normalized using SCTransform and integrated. The first 40 dimensions were used for the initial clustering. The macrophage/monocyte cells as annotated by SingleR were then selected, split into individual samples and integrated again using 30 dimensions. Louvain clustering resulted in four clusters which were annotated based on the expression of marker genes. This integrated dataset served as the reference to map the macrophages/monocytes from SARS-CoV-2 infected BAL using the FindTransferAnchors and MapQuery with reference.reduction set to pca and umap as the reduction.model.

The BAL samples were also annotated using SingleR library with the IM and AM bulk sorted cells as reference. In order to obtain references for assigning cell types in single-cell data, bulk RNA-Seq data of interstitial (IM) and alveolar macrophages (AM) from three uninfected cynomolgus macaques^35^ was analyzed using DESeq2. The regularized log expression values were obtained using the rlog function with the parameters blind = FALSE and filtType = “parametric.” The significant genes were filtered based on following criteria: *p*adj <0.05; fold-change >2 and normalized mean expression >5000 for either IM or AM samples.

For analysis of human lung data, we obtained the rds object for GEO GSE135893^40^ and filtered all cells except those annotated as Macrophages, Monocytes and Proliferating macrophages from Control samples. There were a total of ten control samples from two different sites. We observed some potential batch effects in UMAP and selected only seven samples from the “Vanderbilt” site. We further dropped one sample as the number of macrophages/monocytes was low resulting in a total of six healthy samples. The object was split based on the Sample_Name and reintegrated using CCA method in Seurat. Based on the expression of markers genes described in Morse et al.^41^, the four seurat clusters obtained by using 15 dimension and a resolution of 0.1, were annotated as FABP4hi, SPP1hi, FCN1hi and Proliferating macrophages. The rhesus and human macrophage/monocytes from healthy individuals were then integrated using the reference-based approach with human samples as the reference using genes that were classified as one-to-one orthologs according to ENSEMBL between GRCh38 and Mmul10 and shared the same gene name. The UCell (v1.3.1)^72^ package was used to obtain enrichment scores for marker gene expression between rhesus and human macrophage/monocyte subsets. The markers for each subset were obtained using the FindMarkers function in Seurat for each species using the MAST method and filtered to include those with an adjusted *p* value <0.05 and fold-change of >1.5. These were further filtered to only include genes that were classified as one-to-one orthologs and shared the same gene name between GRCh38 and Mmul10 (Supplementary Data 6).

For human BAL, we used the samples available as part of GEO: GSE145926^8^. The cells were filtered based on the following criteria: nFeature_RNA > 100, nFeature_RNA < 3500 and percent.mito <10 and the samples were integrated using reciprocal PCA. The cells were annotated using BPEncode database in SingleR and only the cells annotated as macrophages/monocytes in the largest cluster comprising of macrophages/monocytes was used for further analysis (Supplementary Fig. 8d, e). These cells were then annotated using the healthy lung macrophage/monocyte as reference using the FindTransferAnchors and MapQuery functions in seurat. The expression of marker genes was used to assess the accuracy of the predictions (Supplementary Fig. 8f).

To calculate the contribution of each cell type toward expression of a gene, the CPM values were obtained using RC normalization method with a scale factor of 1e6. The total CPM value was calculated per gene and the sum of CPM values for a given cell type was divided by the total to obtain a percentage value.

### Mesoscale cytokine analysis

U-PLEX assays (Meso Scale MULTI-ARRAY Technology) were used for plasma and BALF cytokine detection according to manufacturer’s instructions, using 25 microliters as input.

### Statistics and reproducibility

No statistical method was used to predetermine sample size. The sample size was largely determined by (1) availability of NHP that, at the time of the study (March 2020), could be infected with SARS-CoV-2 and housed in BSL-3 and (2) anticipated strong impact of baricitinib in blocking SARS-CoV-2 induced inflammation. The 8 RMs from Cohort 1 were randomized 2 days after infection into a treated and untreated group each comprising of four RMs. Cohort 2 was comprised of 6 SARS-CoV-2-infected RMs^26^. Nasal sgRNA viral loads at 2 dpi were not measured for 4 animals (*n* = 2 Cohort 1 and *n* = 2 baricitinib cohort) and throat sgRNA viral loads at 6 dpi and 8 dpi were not measured for one Cohort 1 animal due to limited RNA. Whole blood was not stained for flow cytometry at 1 dpi for one Cohort 1 and two baricitinib animals and 6 dpi for one Cohort 1 animal. BAL fluid supernatant was not collected for one Cohort 1 and one baricitinib animal at 2 dpi and subsequently not run for mesoscale analysis. One scRNA-Seq baseline sample from cohort 2 was dropped due to large fraction of epithelial cells. The Investigators were not blinded to allocation during experiments and outcome assessment. Statistical tests were performed using R (version 4.2.2) or GraphPad Prism v7.02 and have been listed accordingly. The specific tests that were used: one-tailed/two-tailed, Mann–Whitney U/Wilcoxon signed-rank test have been indicated for each comaprison. For differential gene expression analysis of bulk and single-cell RNA-Seq data, the adjusted *p* values after multiple test correction, determined as part of DESeq2 and MAST analyses were used.

### Reporting summary

Further information on research design is available in the Nature Portfolio Reporting Summary linked to this article.

## Supplementary information

Supplementary Information

Description of Additional Supplementary Files

Supplementary Data 1

Supplementary Data 2

Supplementary Data 3

Supplementary Data 4

Supplementary Data 5

Supplementary Data 6

Reporting Summary

## Data Availability

The bulk RNA-Seq data generated in this study for 7 dpi and 10 dpi/11 dpi samples for BAL and −5 dpi, 1 dpi, 2 dpi, 4 dpi, 6 dpi, 7 dpi, 8 dpi and 10/11 dpi for PBMC has been deposited in NCBI GEO (GSE198882). The scRNA-Seq data for BAL from SARS-CoV-2 infected rhesus macaques and the bulk RNA-Seq data for −5 dpi, 2 dpi and 4 dpi for bulk were obtained from GEO GSE159214^20^. The 10X single-cell uninfected rhesus macaque lung samples were obtained from GEO GSE149758^33^. The bulk RNA-Seq data for sorted interstitial and alveolar macrophages from cynomolgus macaque were obtained from GEO GSE225316^35^. The single-cell uninfected human lung samples and the human BAL samples were obtained from GEO GSE135893^40^ and GSE145926^8^ respectively. Source data are provided with this paper.

## Source data

Source Data

## Code availability

The scripts used for analysis are available at https://github.com/BosingerLab/NHP_COVID-19_2^73^.
