# Supplementary material for: TREM2+ and interstitial-like macrophages orchestrate airway inflammation in SARS-CoV-2 infection in rhesus macaques
Source: Nat Commun. 2023 Apr 6;14:1914. doi: 10.1038/s41467-023-37425-9 (PMC10078029; doi:10.1038/s41467-023-37425-9)
Supplement: Supplementary file 1 — Supplementary Information [file 41467_2023_37425_MOESM1_ESM.pdf]

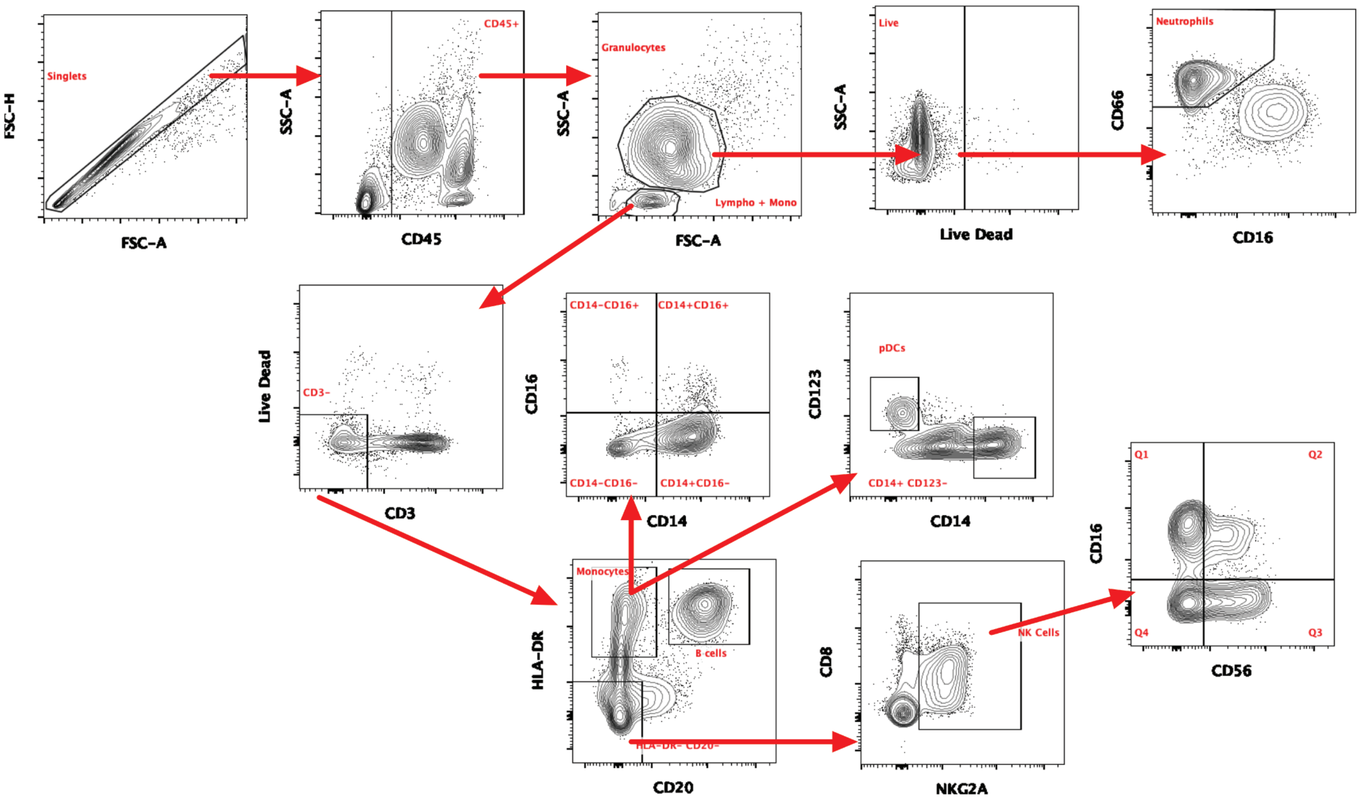

Supplementary Figure 1. Flow gating strategy for different immune cell populations. Representative flow cytometry gating strategy for the longitudinal immunophenotyping of fresh whole blood and cells isolated from BAL. Plots shown are for whole blood staining. Gates are depicted in black boxes with red arrows indicating the gating hierarchy.

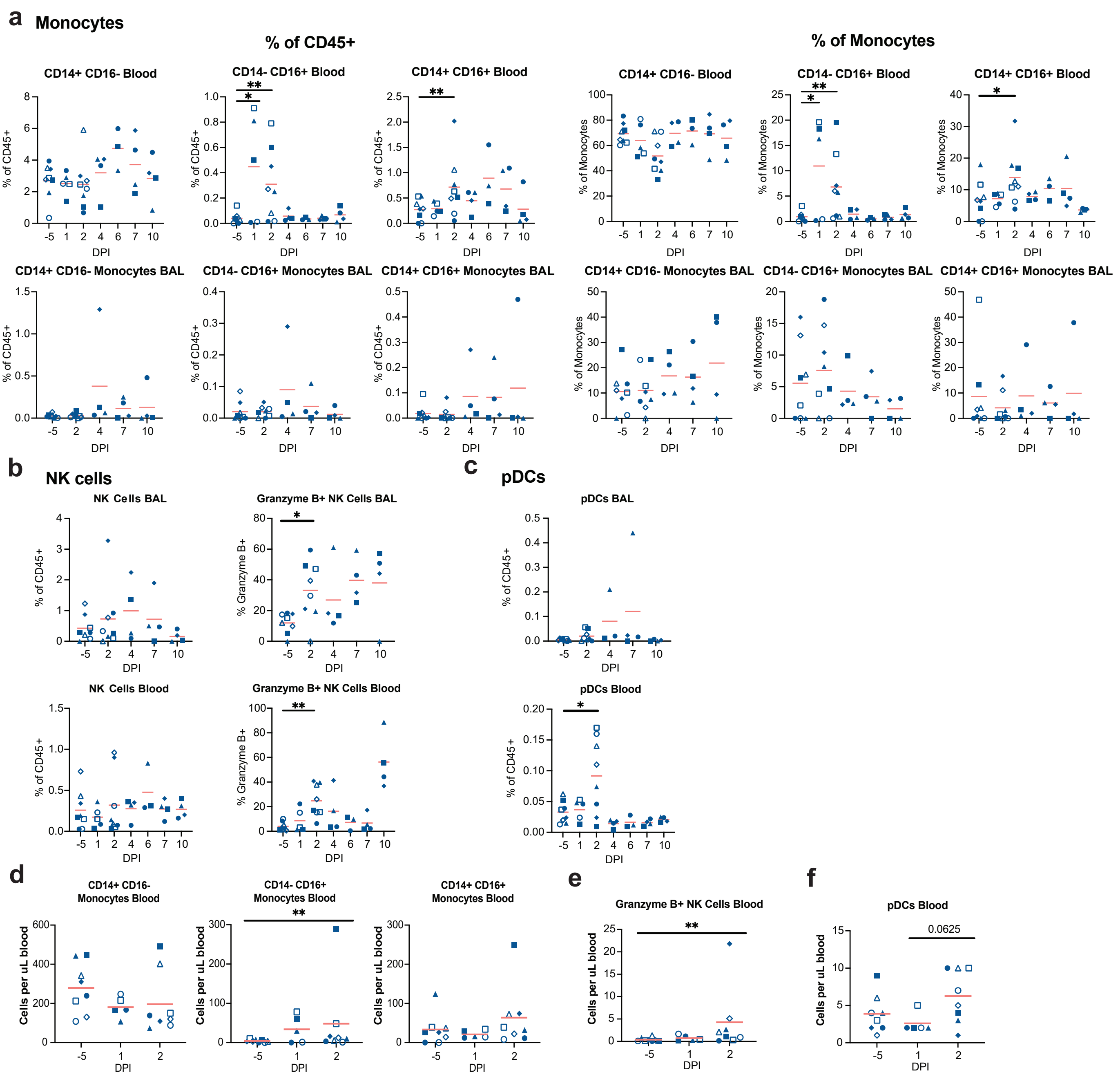

Supplementary Figure 2. Longitudinal flow cytometric analysis in BAL and blood following SARS-CoV-2 infection. n = 8 RM from Cohort 1 (n=4) and baricitnib cohort (n=4) for -5dpi and 2dpi, n= 5 RM from Cohort 1 (n=3) and baricitinib cohort (n=2) for 1dpi blood samples and n=4 RM from Cohort 1 at 4dpi, 7dpi, and 10dpi, and n= 3 RM from Cohort 1 at 6dpi. Open symbols represent baricitnib cohort that started receiving treatment at 2dpi and the filled symbols represent Cohort 1 that were untreated. **(a)** Longitudinal levels of monocytes within BAL and blood depicted as the percentage of CD45+ cells and percentage of monocytes (CD3- CD20- HLA-DR+). p-values for percentage of CD45+: CD14-CD16+ monocytes blood: 1 dpi vs -5 dpi = 0.03 and 2 dpi vs -5 dpi = 0.004; CD14+CD16+ monocytes blood 2 dpi vs -5 dpi = 0.004. p-values for percentage of monocytes: CD14-CD16+ monocytes blood: 1 dpi vs -5 dpi = 0.03 and 2 dpi vs -5 dpi = 0.004; CD14+CD16+ monocytes blood 2 dpi vs -5 dpi = 0.02 **(b)** Longitudinal levels of NK cells as a percentage of CD45+ cells and frequency of NK cells expressing Granzyme B in BAL and blood. p-values for Granzyme B+ NK Cells 2 dpi vs -5 dpi BAL = 0.01 and blood = 0.008. **(c)** Longitudinal levels of plasmacytoid dendritic cells (pDCs) within BAL and blood depicted as a percentage of CD45+ cells. **(d, e, f)** Absolute counts of monocytes (d), Granzyme B expressing NK cells (e) and pDCs (f).p-values for (d) CD14-CD16+ monocytes in blood 2 dpi vs -5 dpi = 0.008, (e) Granzyme B+ NK cells in blood 2 dpi vs -5 dpi = 0.008. The red bars represent the mean. Statistical analysis was performed using one-tailed Wilcoxon signed-rank test comparing each time point to -5dpi (a-c) and two-tailed Wilcoxon test (d-f) in GraphPad Prism v7.02 . * p-value < 0.05, ** p-value < 0.01. Source data (a-f) are provided as a Source Data file.

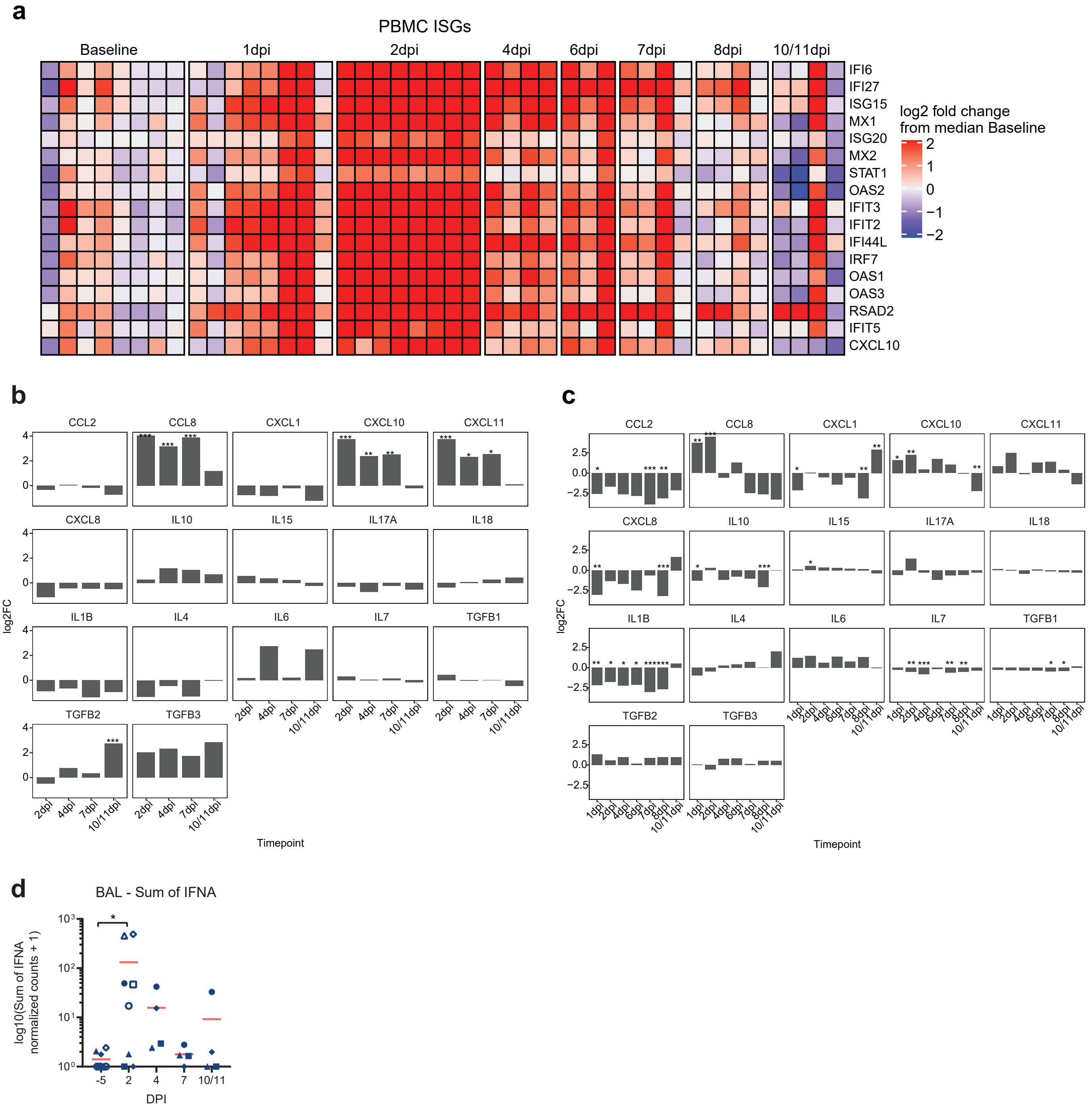

Supplementary Figure 3. Bulk transcriptomic analysis of airways and peripheral blood. n = 8 RM from Cohort 1 (n=4) and baricitnib cohort (n=4) for -5dpi, 1dpi and 2dpi, n= 4 RM from Cohort 1 starting at 4dpi. **(a)** Heatmap showing expression of ISG in PBMC over all sampled time points. The color scale indicates log2 expression relative to the median of -5dpi samples. **(b & c)** Normalized expression of cytokines and chemokines in bulk RNA-Seq in BAL (b) and PBMC(c). Statistical analysis was performed using DESeq2. The p-values corrected by the default Benjamini and Hochberg method were used. * padj < 0.05, ** padj < 0.01, *** padj < 0.001. The exact p-values are included in Supplementary Data 2 and 3. **(d)** Sum of normalized expression of IFNA in longitudinal BAL samples * p-value = 0.04. The red bars represent the mean. Statistical analysis was performed using one-tailed Wilcoxon signed-rank test in R v4.2.2 comparing each time point to -5dpi. * p-value < 0.05. Source data (d) are provided as a Source Data file.

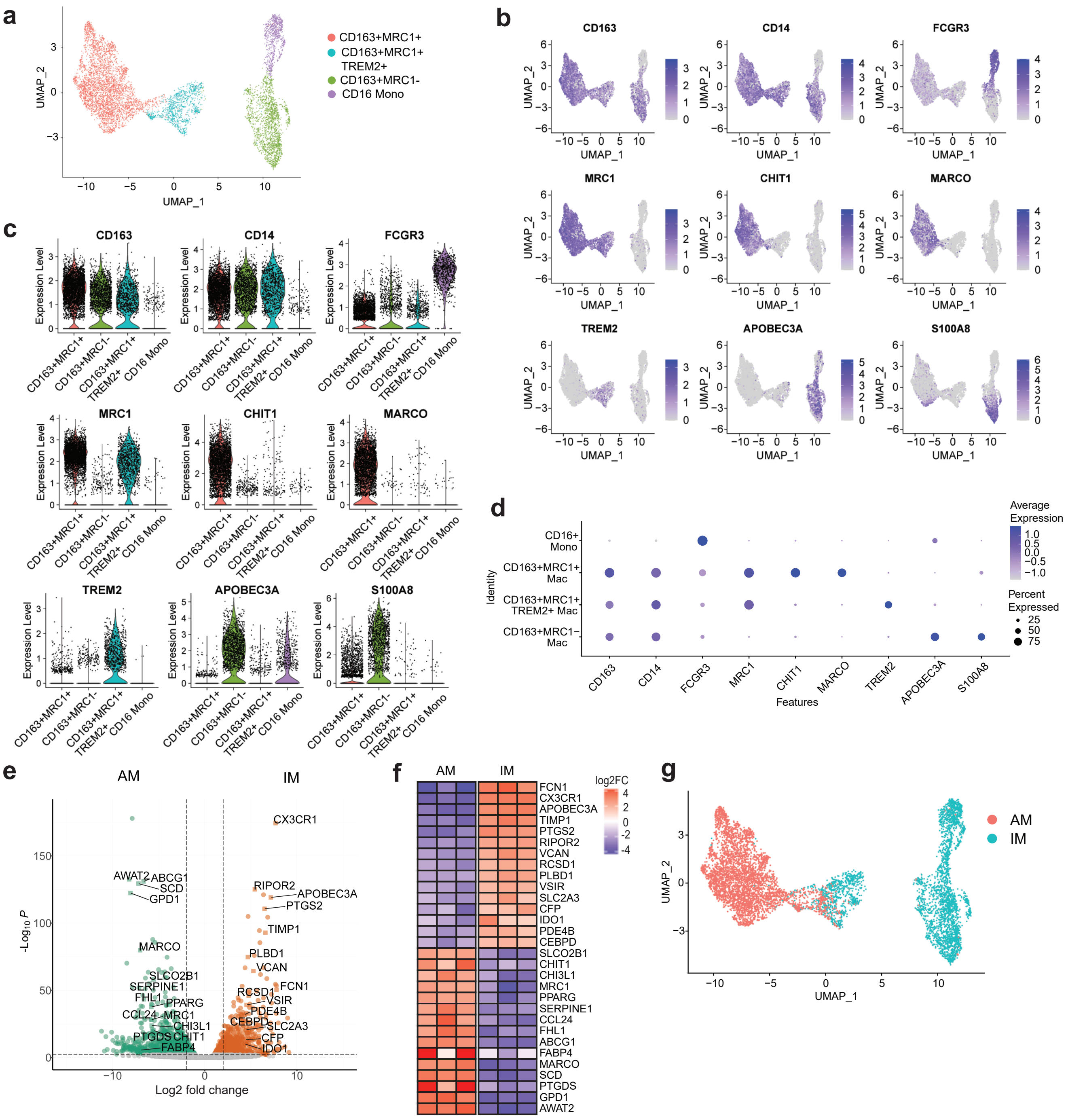

Supplementary Figure 4. References used for annotating macrophage/monocyte subsets. (a) UMAP of macrophages/monocytes from 10X lung samples of three uninfected rhesus macaques (NCBI GEO: GSE1497581) showing Louvain clustering. **(b & c)** FeaturePlot (b) and Violin Plots (c) showing the expression of marker genes in the macrophage/monocyte clusters. **(d)** DotPlot showing expression of marker genes for the different monocyte/macrophage subsets as defined previously[1,2]. **(e)** Volcano plots showing differentially expressed genes for pairwise comparison of alveolar (n=3) and interstitial macrophages (n=3)[3]. The thresholds used are an adjusted p-value < 0.05 and a fold change of 2 for alveolar vs interstitial macrophage. Top 15 genes that have a mean normalized expression of at least 5000 for either type have been indicated. Statistical analysis was performed using DESeq2. The p-values corrected by the default Benjamini and Hochberg method were used. **(f)** Heatmap showing the top 15 genes for each subset. The color scale indicates log2 expression relative to the median of all samples. **(g)** UMAP of single-cell 10X lung samples showing SingleR annotations using the bulk sorted cells as reference.

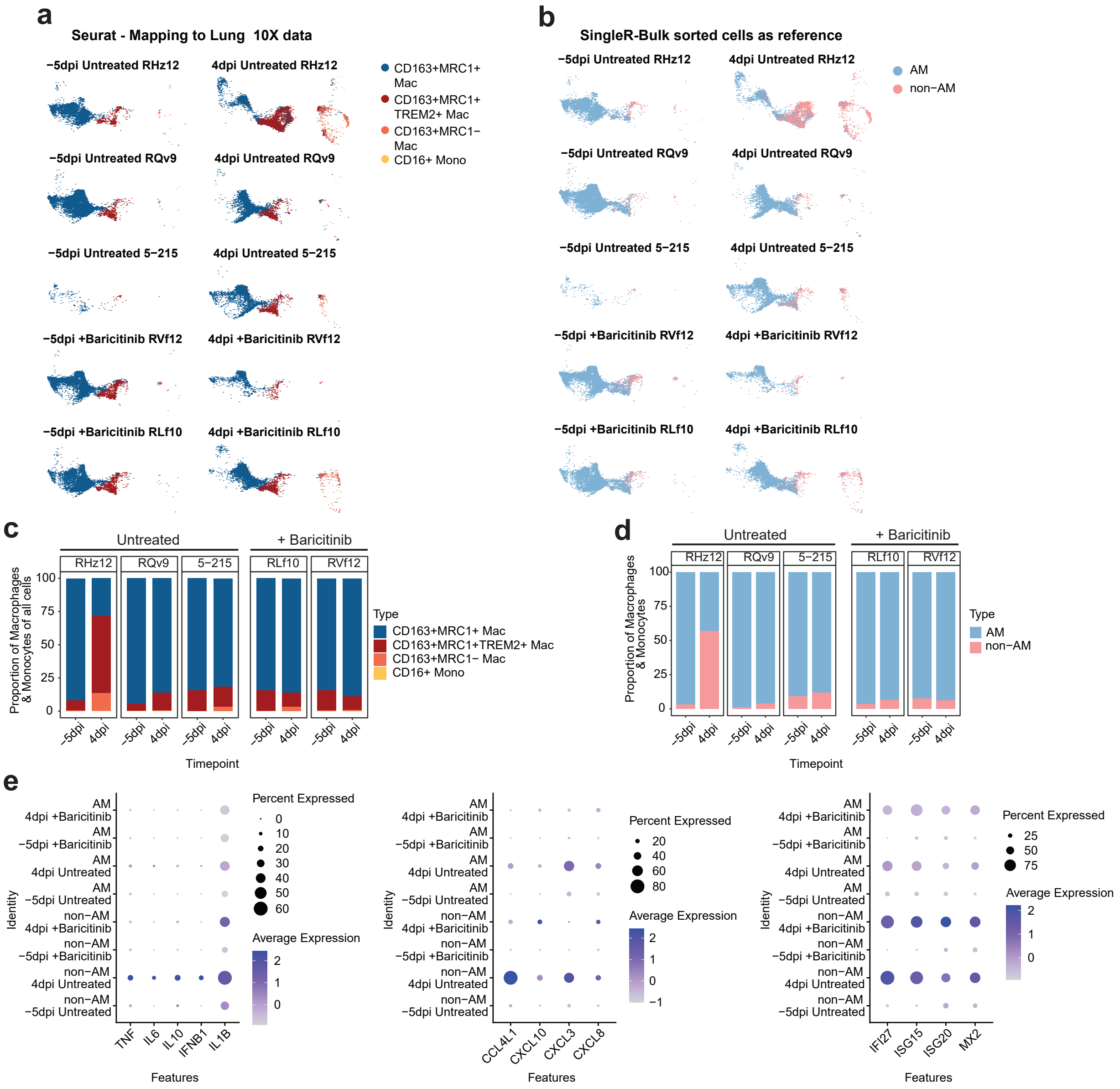

Supplementary Figure 5. Cell annotation using bulk sorted cells as reference. (a & b) The BAL macrophage/monocytes from SARS-CoV2 infected rhesus macaques (three untreated - Cohort 1 and two baricitinib treated) projected on the 10X lung reference macrophages/monocytes UMAP split by each animal and timepoint. **(a)** Annotations predicted from mapping to 10X lung samples using Seurat **(b)** Annotations predicted by SingleR using the bulk sorted AM and IM cells as reference. **(c & d)** Percentage of a macrophage/monocytes subset out of all the macrophage/monocyte cells in a given sample based on 10X lung reference (c) and the bulk sorted cells as reference (d). **(e)** DotPlots showing expression of pro-inflammatory cytokines, chemokines and ISG in macrophage/monocyte subsets based on the bulk sorted cells reference at -5dpi and 4dpi in BAL samples from untreated (Cohort 1) and baricitinib treated rhesus macaques. Source data (c,d) are provided as a Source Data file.

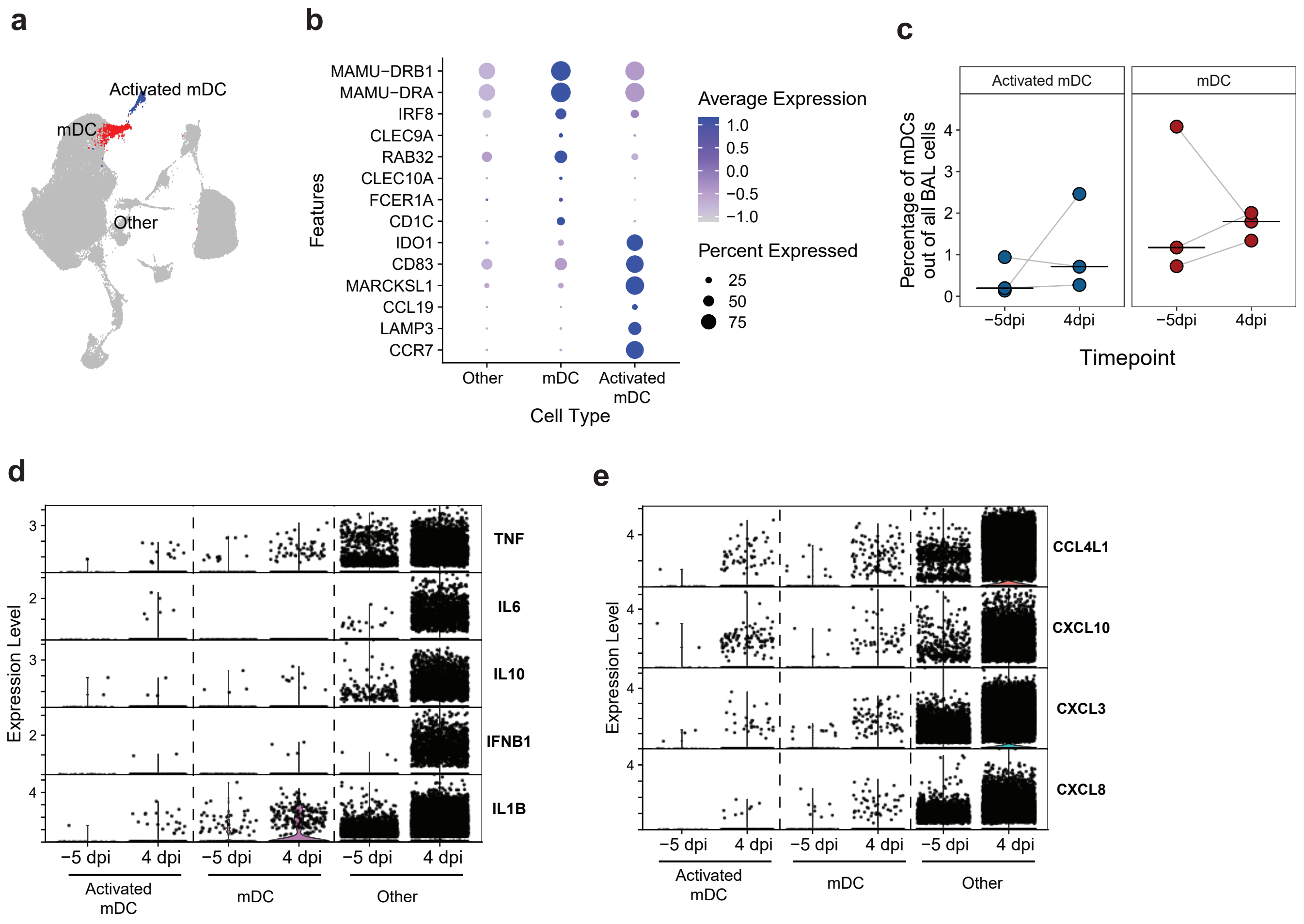

Supplementary Figure 6. Expression of cytokines and chemokines in mDC from rhesus macaque BAL. n = 3 RM from Cohort 1 for both -5dpi and 4dpi. **(a)** UMAP showing clusters annotated as mDC and activated mDC in integrated data comprising of untreated (Cohort 1) and baricitnib treated animals. **(b)** Dot plot showing the expression of marker genes used for annotation of mDC clusters from untreated (Cohort 1) and baricitnib treated animals. **(c)** Percentage of mDCs out of all the BAL cells in untreated animals (Cohort 1). The black lines indicate the median. **(d & e)** Violin plots showing the expression of cytokines (d) and chemokines (e) in mDC subsets compared to all other BAL cells from untreated animals. Source data (c) are provided as a Source Data file.

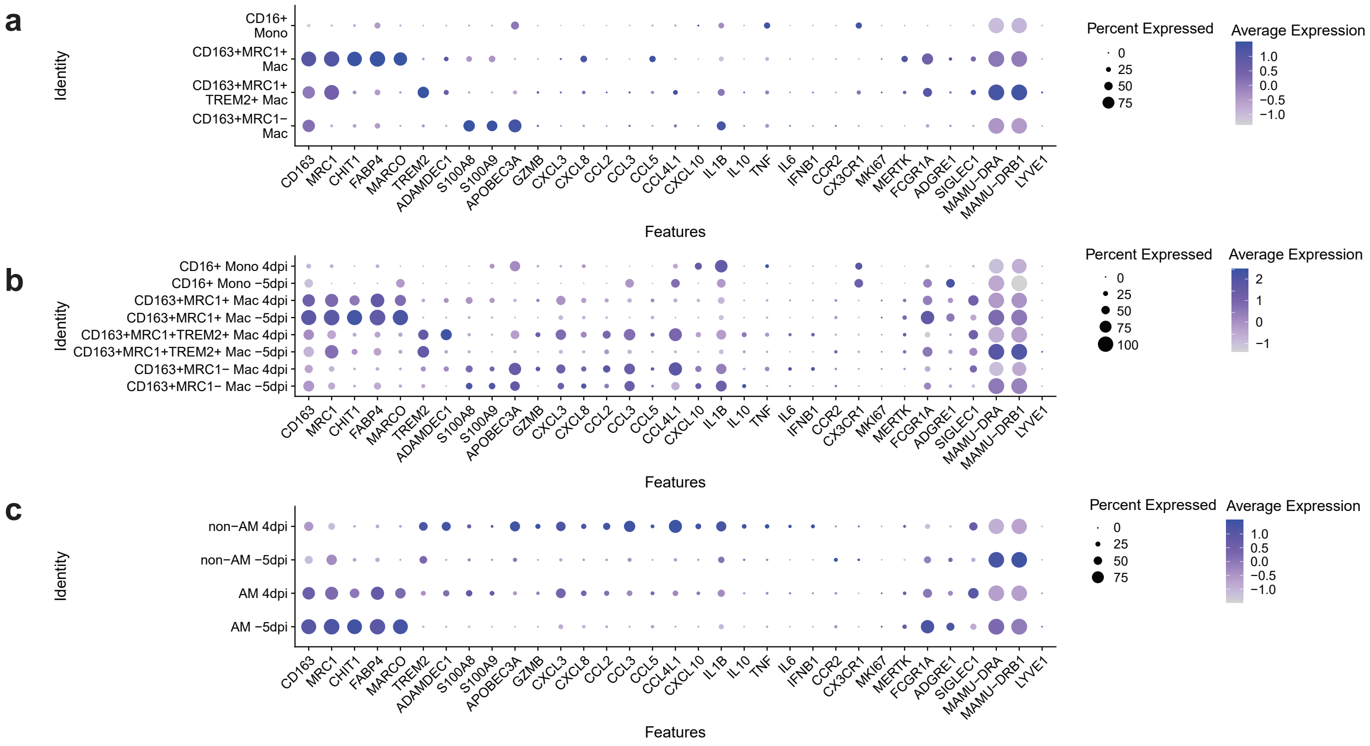

Supplementary Figure 7. Expression of canonical markers and pro-inflammatory genes in different macrophage/monocyte subsets. (a) 10X lung control samples from three uninfected RM. **(b & c)** BAL samples from three SARS-CoV2 infected RM (Cohort 1) annotated using the 10X lung reference (b) or the bulk sorted cells as reference (c).

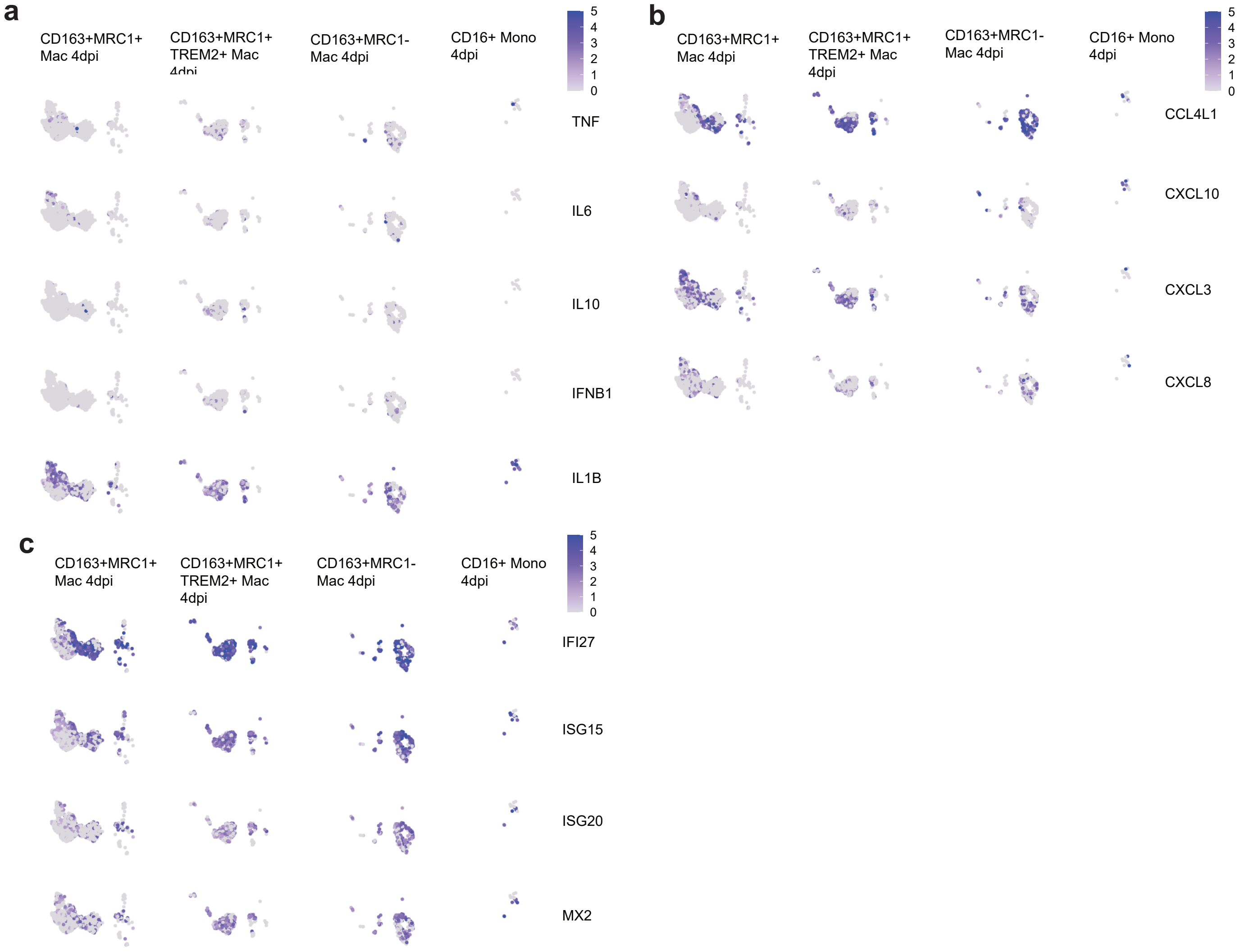

Supplementary Figure 8. Expression of pro-inflammatory mediators in rhesus macaque BAL macrophages/monocytes at 4 dpi (Cohort 1 n= 3). Feature plots showing expression of **(a)** cytokines, **(b)** chemokines, and **(c)** ISG in the different macrophage/monocyte subsets from rhesus macaque BAL.

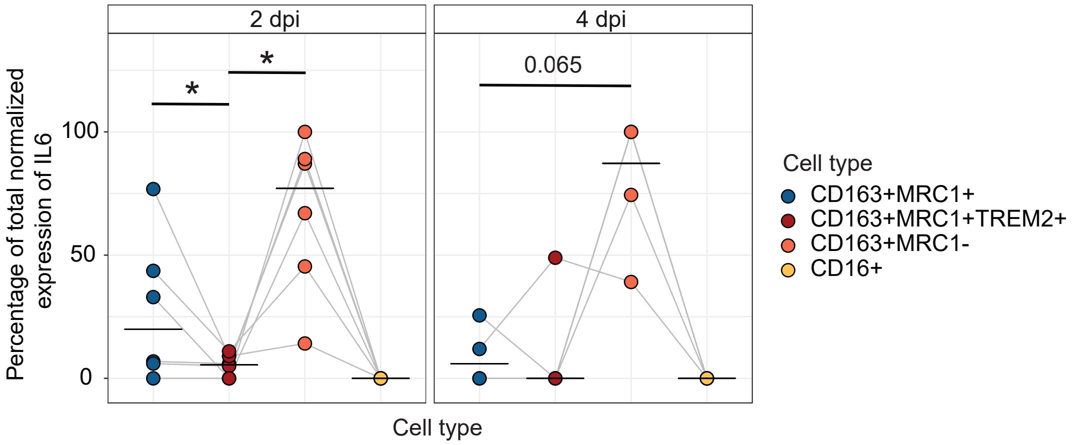

Supplementary Figure 9. Contribution of each macrophage/monocyte subset towards the production of IL6 at 2 dpi and 4 dpi in Cohort 2 (n = 6). The percentage was obtained by summing the normalized expression of IL6 for each subset and dividing by the total normalized expression of IL6 from all subsets separately for each time point. The black lines indicate the median. Statistical test was performed using two-tailed Wilcoxon signed rank test in R v4.2.2. p-values: 2dpi CD163+MRC1+ vs CD163+MRC1+TREM2+ = 0.04 and CD163+MRC1+TREM2+ vs CD163+MRC1- = 0.03. * p-value < 0.05. Source data are provided as a Source Data file.

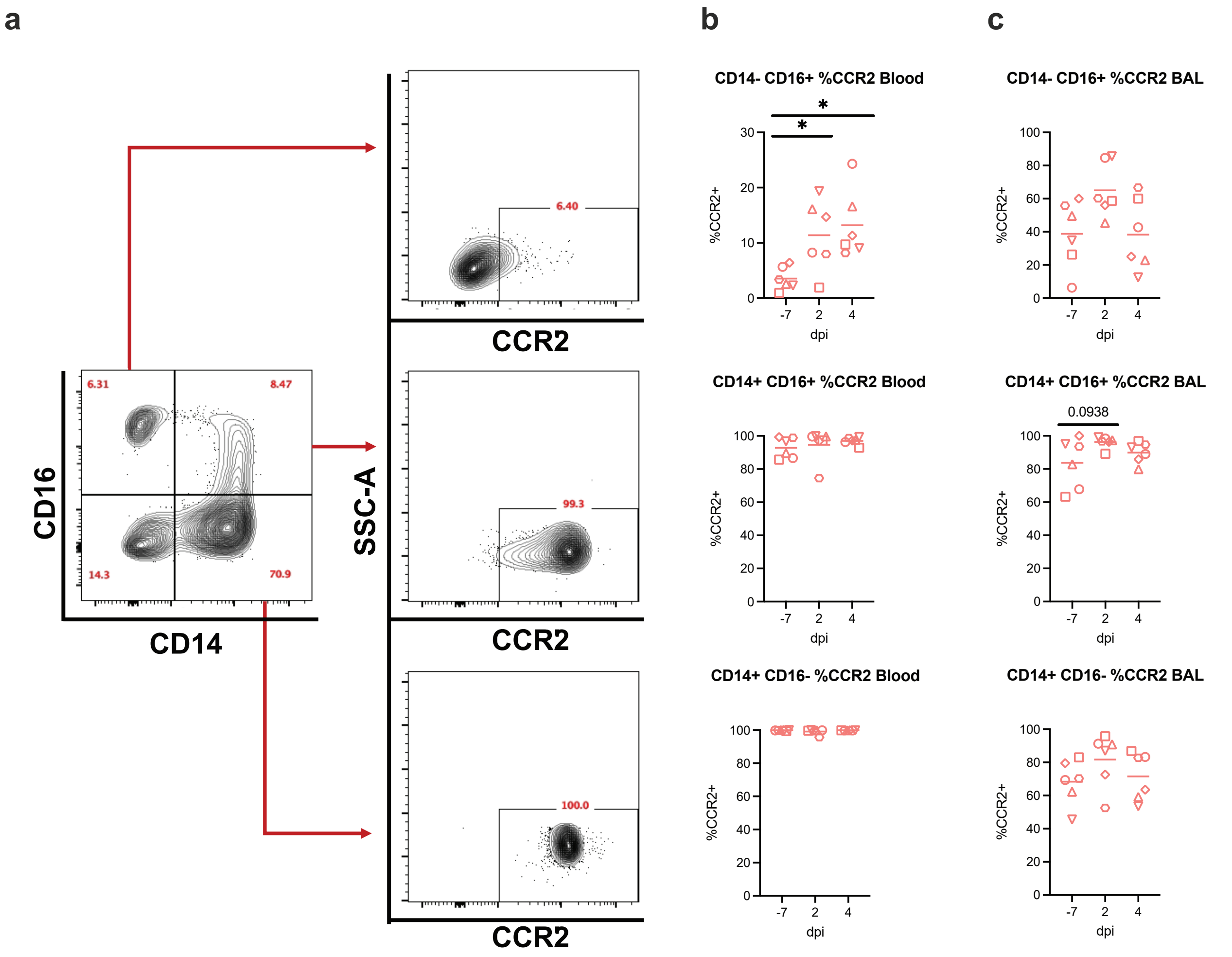

Supplementary Figure 10. Expression of CCR2 in monocytes in rhesus macaque BAL (Cohort 2 n = 6) (a) Representative Flow plots (b) Percentage of CCR2+ monocytes in blood (c) Percentage of CCR2+ monocytes in BAL. The red bar represents the mean. Statistical analysis was performed using two-tailed Wilcoxon signed-rank test in GraphPad Prism v7.02 comparing each time point. * p-value = 0.0312 . Source data (b,c) are provided as a Source Data file.

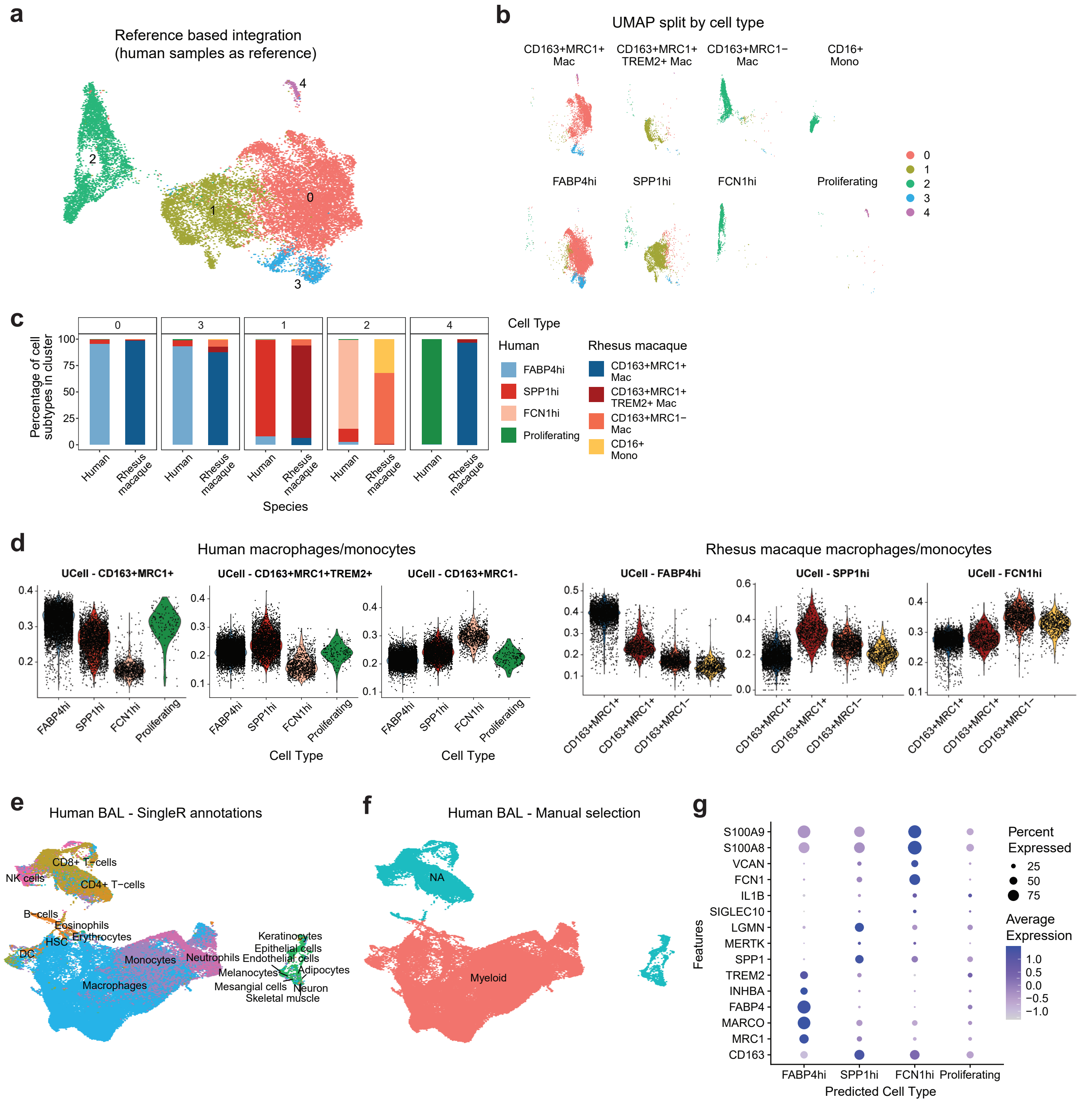

Supplementary Figure 11. Comparison of human lung macrophage/monocytes subsets with that of rhesus macaque (a) UMAP of macrophages/monocytes from lung samples of six healthy human donors[4] and three healthy rhesus macaques[1] integrated with the human samples as reference using one-to-one orthologs between GRCh38 and Mmul10 with shared gene names. **(b)** UMAP from (a) split by the cell types. The top row corresponds to the rhesus macrophages/monocytes while the bottom are from human. **(c)** Distribution of different cell types in each seurat cluster. Percentage of a cell type is indicated out of all human or rhesus macrophage/-monocyte subsets in a given cluster. **(d)** Violin plots showing the UCell enrichment score for marker gene expression. The top row shows the enrichment of rhesus macrophage/monocyte marker genes in human macrophages/-monocytes and the bottom row shows the enrichment of human macrophage/monocyte marker genes in rhesus macrophages/monocytes **(e & f)** UMAP of integrated human BAL samples[5] (healthy=3, mild COVID-19 = 3 and severe COVID-19 = 6) colored by SingleR-based annotation (d) or manual selection of the myeloid cluster (e). **(g)** DotPlot indicating expression of marker genes in the predicted cell types in human BAL samples based on mapping to the human healthy lung macrophages/monocytes. Source data (c) are provided as a Source Data file.

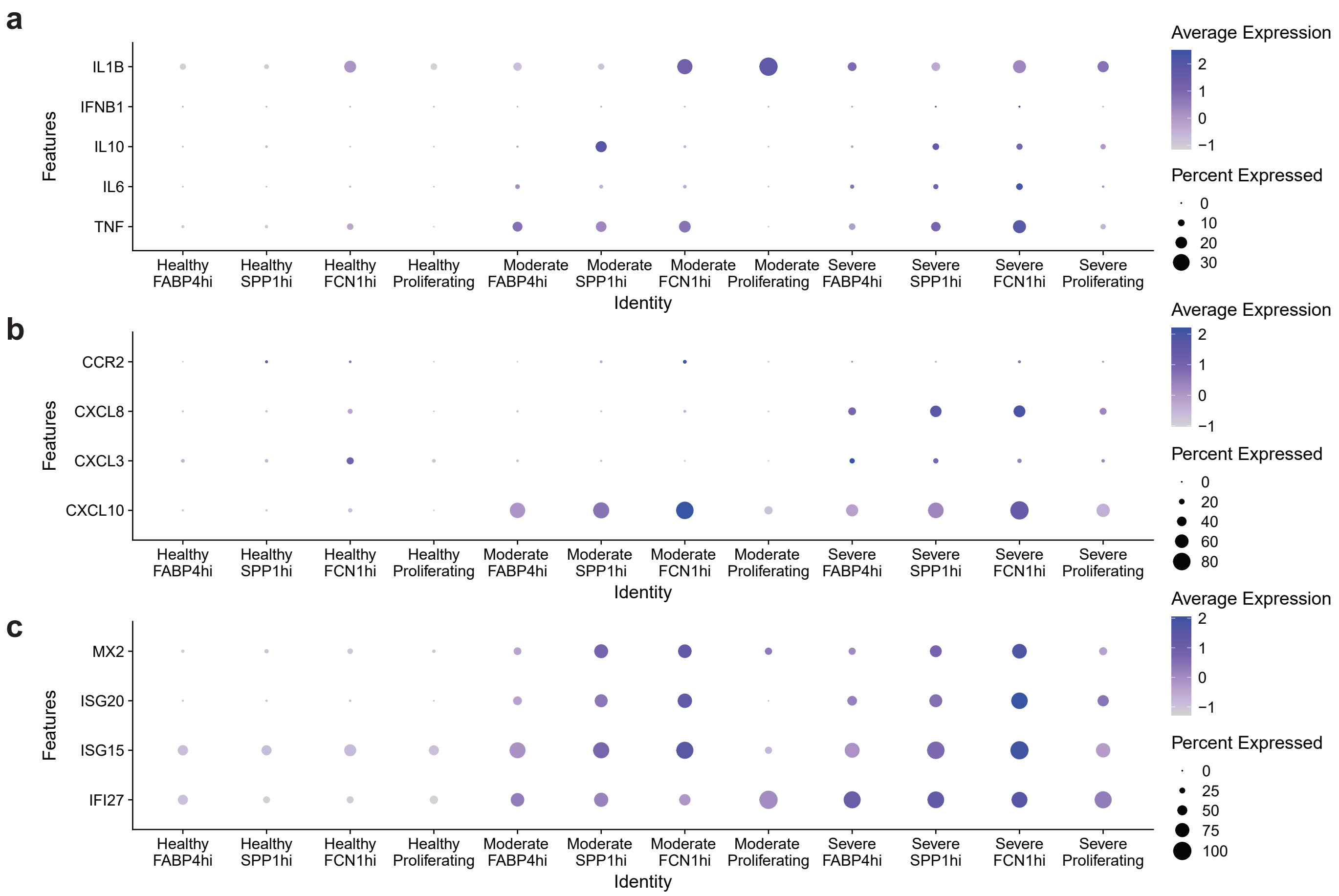

Supplementary Figure 12. Expression of pro-inflammatory mediators in human BAL samples. Expression of **(a)** pro-inflammatory cytokines,**(b)** chemokines and **(c)** ISG in the different macrophage/monocyte subsets predicted in human BAL samples based on mapping to healthy human macrophages/monocytes.

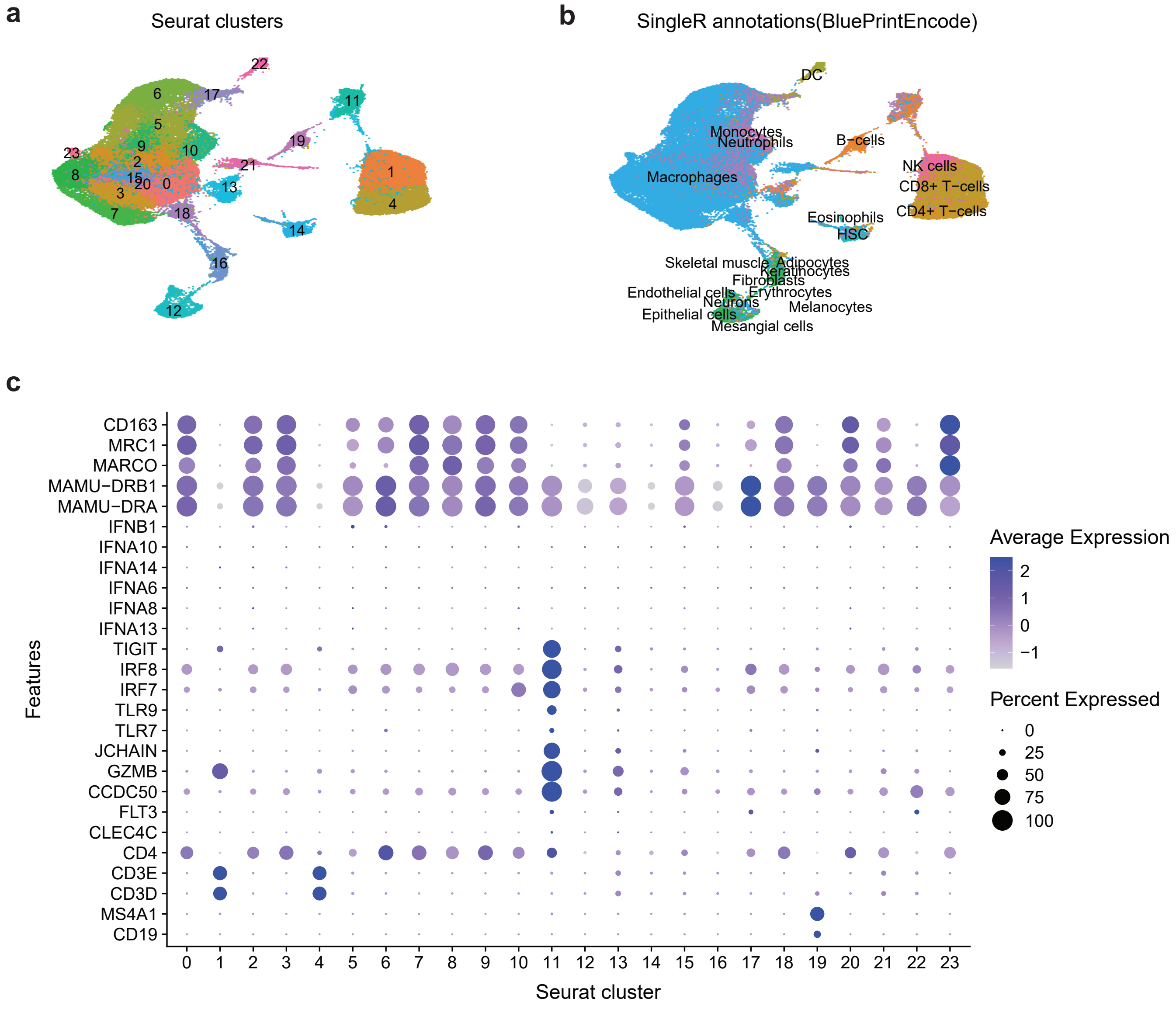

Supplementary Figure 13. Annotation of pDC in BAL from untreated Cohort 1 (n=3) and baricitinib treated (n=2) rhesus macaques. (a & b) UMAP after integration of all five samples colored according to seurat clusters (a) or SingleR-based annotations (b). **(c)** Dot plot showing expression of canonical marker genes for different Seurat clusters for the five rhesus BAL samples. Cluster 11 was annotated as pDC cells.

Supplementary References

1 Esaulova, E. *et al.* The immune landscape in tuberculosis reveals populations linked to disease and latency. *Cell Host Microbe* **29**, 165-178 e168, doi:10.1016/j.chom.2020.11.013 (2021).

2 Cai, Y. *et al.* In vivo characterization of alveolar and interstitial lung macrophages in rhesus macaques: implications for understanding lung disease in humans. *J Immunol* **192**, 2821-2829, doi:10.4049/jimmunol.1302269 (2014).

3 Corry, J. *et al.* Infiltration of inflammatory macrophages and neutrophils and widespread pyroptosis in lung drive influenza lethality in nonhuman primates. *PLoS Pathog* **18**, e1010395, doi:10.1371/journal.ppat.1010395 (2022).

4 Habermann, A. C. *et al.* Single-cell RNA sequencing reveals profibrotic roles of distinct epithelial and mesenchymal lineages in pulmonary fibrosis. *Sci Adv* **6**, eaba1972, doi:10.1126/sciadv.aba1972 (2020).

5 Liao, M. *et al.* Single-cell landscape of bronchoalveolar immune cells in patients with COVID-19. *Nat Med* **26**, 842-844, doi:10.1038/s41591-020-0901-9 (2020).
