# Supplementary material for: TREM2+ and interstitial-like macrophages orchestrate airway inflammation in SARS-CoV-2 infection in rhesus macaques
Source: Nat Commun. 2023 Apr 6;14:1914. doi: 10.1038/s41467-023-37425-9 (PMC10078029; doi:10.1038/s41467-023-37425-9)
Supplement: Supplementary file 2 — Description of Additional Supplementary Files [file 41467_2023_37425_MOESM2_ESM.pdf]

Description of Additional Supplementary Files

- **Supplementary Data 1.** GSEA results for bulk BAL and PBMC samples
- **Supplementary Data 2.** DESeq2 results for bulk BAL samples
- **Supplementary Data 3.** DESeq2 results for bulk PBMC samples
- **Supplementary Data 4.** Differential gene expression analysis for macrophage/monocyte subsets in 10X BAL samples (Cohort 1)
- **Supplementary Data 5.** Cell numbers for each macrophage/monocyte subset
- **Supplementary Data 6**. Marker genes for human and rhesus macrophage/monocyte subsets determined by running FindMarkers function in Seurat separately for each species. Genes were considered significant if fold-change > 1.5 and p-adj < 0.05.
