# Supplementary material for: TREM2+ and interstitial-like macrophages orchestrate airway inflammation in SARS-CoV-2 infection in rhesus macaques
Source: Nat Commun. 2023 Apr 6;14:1914. doi: 10.1038/s41467-023-37425-9 (PMC10078029; doi:10.1038/s41467-023-37425-9)
Supplement: Supplementary file 9 — Reporting Summary [file 41467_2023_37425_MOESM9_ESM.pdf]

nature portfolio

Corresponding author(s): Steven Bosinger
Mirko Paiardini

Last updated by author(s): 02/16/2023

Reporting Summary

Nature Portfolio wishes to improve the reproducibility of the work that we publish. This form provides structure for consistency and transparency in reporting. For further information on Nature Portfolio policies, see our Editorial Policies and the Editorial Policy Checklist.

Statistics

For all statistical analyses, confirm that the following items are present in the figure legend, table legend, main text, or Methods section.

n/a	Confirmed	
☐	☒	The exact sample size (*n*) for each experimental group/condition, given as a discrete number and unit of measurement
☐	☒	A statement on whether measurements were taken from distinct samples or whether the same sample was measured repeatedly
☐	☒	The statistical test(s) used AND whether they are one- or two-sided<br>*Only common tests should be described solely by name; describe more complex techniques in the Methods section.*
☐	☒	A description of all covariates tested
☐	☒	A description of any assumptions or corrections, such as tests of normality and adjustment for multiple comparisons
☐	☒	A full description of the statistical parameters including central tendency (e.g. means) or other basic estimates (e.g. regression coefficient) AND variation (e.g. standard deviation) or associated estimates of uncertainty (e.g. confidence intervals)
☐	☒	For null hypothesis testing, the test statistic (e.g. *F*, *t*, *r*) with confidence intervals, effect sizes, degrees of freedom and *P* value noted<br>*Give P values as exact values whenever suitable.*
☒	☐	For Bayesian analysis, information on the choice of priors and Markov chain Monte Carlo settings
☒	☐	For hierarchical and complex designs, identification of the appropriate level for tests and full reporting of outcomes
☒	☐	Estimates of effect sizes (e.g. Cohen's *d*, Pearson's *r*), indicating how they were calculated

Our web collection on statistics for biologists contains articles on many of the points above.

Software and code

Policy information about availability of computer code

Data collection	Flow Cytometry data were collected using FACS DiVa software 8.0 (BD Biosciences) on a FACS Symphony. Cytokine data was collected using MSD systems. The bulk RNA-Seq libraries were sequenced on an Illumina NovaSeq6000.
Data analysis	Flow Cytometry data were processed using FlowJo version 10.7 (TreeStar). bcl2fastq v2.20.0.422 used to convert BCL to Fastq files. STAR v2.7.3a was used to align reads to a composite reference of Macaca mulatta (Mmul10 Ensembl release 100), SARS-CoV-2 (strain MN985325.1 - NCBI) and ERCC sequences. DESeq2 v1.24.0, GSEA v4.1.0, EnhancedVolcano v1.8.0, and ComplexHeatmap v2.0.0 were used for analysis of bulk RNA-Seq data. Seurat v4.0.4, SingleR v1.4.0, MAST v1.16.0, and UCell v1.3.1 were used for analyzing scRNA-Seq data. The R scripts used for analyzing data are available at https://github.com/BosingerLab/NHP_COVID-19_2. Statistics were performed using GraphPad Prism version 7.02 and R v4.2.2.

For manuscripts utilizing custom algorithms or software that are central to the research but not yet described in published literature, software must be made available to editors and reviewers. We strongly encourage code deposition in a community repository (e.g. GitHub). See the Nature Portfolio guidelines for submitting code & software for further information.

Data

Policy information about availability of data

All manuscripts must include a data availability statement. This statement should provide the following information, where applicable:

- Accession codes, unique identifiers, or web links for publicly available datasets
- A description of any restrictions on data availability
- For clinical datasets or third party data, please ensure that the statement adheres to our policy

The bulk RNA-Seq data generated in this study for 7dpi and 10dpi/11dpi samples for BAL and -5dpi, 1dpi, 2dpi, 4dpi, 6dpi, 7dpi, 8dpi and 10/11dpi for PBMC has been deposited in NCBI GEO (GSE198882). The scRNA-Seq data for BAL from SARS-CoV-2 infected rhesus macaques and the bulk RNA-Seq data for -5dpi, 2dpi and 4dpi for bulk were obtained from GEO GSE15921420. The 10X single-cell uninfected rhesus macaque lung samples were obtained from GEO GSE14975833. The bulk RNA-Seq data for sorted interstitial and alveolar macrophages from cynomolgus macaque were obtained GEO GSE22531635. The single-cell uninfected human lung samples and the human BAL samples were obtained from GEO GSE13589340 and GSE1459268 respectively. Source data for the figures are provided with the paper.

Human research participants

Policy information about studies involving human research participants and Sex and Gender in Research.

Reporting on sex and gender	N/A
Population characteristics	N/A
Recruitment	N/A
Ethics oversight	N/A

Note that full information on the approval of the study protocol must also be provided in the manuscript.

Field-specific reporting

Please select the one below that is the best fit for your research. If you are not sure, read the appropriate sections before making your selection.

☒ Life sciences ☐ Behavioural & social sciences ☐ Ecological, evolutionary & environmental sciences

For a reference copy of the document with all sections, see nature.com/documents/nr-reporting-summary-flat.pdf

Life sciences study design

All studies must disclose on these points even when the disclosure is negative.

Sample size	Cohort 1 (n=4) were infected with SARS-CoV-2. Baricitinib cohort (n=4) were infected with SARS-CoV-2 after day 2 post infection were treated with baricitinib. We compared the parameters of interest longitudinally in the same animals prior to and during infection the treatment as well as between different groups.<br>Cohort 2: n= 6 additional animals recruited to study CCR2 flow cytometry experiments in monocytes and for scRNA-seq dataset.<br><br>Sample size calculation was not performed, and largely determined by (i) availability of NHP that, at the time of the study (March 2020), could be infected with SARS-CoV-2 and housed in BSL-3 and (ii) anticipated strong impact of baricitinib in blocking SARS-CoV-2 induced inflammation.
Data exclusions	Nasal sgRNA viral loads at 2dpi were not measured for 4 animals (n=2 Cohort 1 and n=2 baricitinib cohort) and throat sgRNA viral loads at 6dpi and 8dpi were not measured for one Cohort 1 animal due to limited RNA. Whole blood was not stained for flow cytometry at 1dpi for one Cohort 1 and two baricitinib animals and 6dpi for one Cohort 1 animal. BAL fluid supernatant was not collected for one Cohort 1 and one baricitinib animal at 2dpi and subsequently not run for mesoscale analysis. One baseline scRNA-Seq sample from Cohort 2 was not used as it had unusually large fraction of epithelial cells.
Replication	Initially, we performed analyses in 8 RMs infected with 1.1x106 PFU of the USA-WA1/2020 strain of SARS-CoV-2, 4 of which were untreated (Cohort 1) and 4 that were baricitinib-treated. These 8 animals were divided into 3 groups of 2-3 animals each for infection; each group including at least one control and baricitinib-treated RM. To ensure that our findings in infected, untreated animals were robust, we performed the same analyses in 6 additional animals (Cohort 2) that were enrolled in a study investigating the role of Type I IFN in SARS-CoV-2-infection (PMID: 36324810). Cohort 2 animals were also infected with 1.1x106 PFU of the USA-WA1/2020 strain of SARS-CoV-2, divided equally into 3 infection groups, and remained untreated during the duration of the study. As a result, infection was performed in a total of 6 different experimental replicates, with similar findings in untreated RMs being detected across all infection groups. No attempts were made to replicate the data in baricitinib-treated RMs due to substantial costs pertaining to animal acquisition and care.
Randomization	Animals were stratified into treatment groups based on acute (“peak”) viral load, sex and age.

Blinding	Blinding of primary investigators was not possible as we were responsible of the distribution of baricitinib for administration and stratification of the animals into treatment groups. Secondary collaborators were blinded to treatment groups prior to analysis.

Reporting for specific materials, systems and methods

We require information from authors about some types of materials, experimental systems and methods used in many studies. Here, indicate whether each material, system or method listed is relevant to your study. If you are not sure if a list item applies to your research, read the appropriate section before selecting a response.

Materials & experimental systems

n/a	Involved in the study
☐	☒ Antibodies
☒	☐ Eukaryotic cell lines
☒	☐ Palaeontology and archaeology
☐	☒ Animals and other organisms
☒	☐ Clinical data
☒	☐ Dual use research of concern

Methods

n/a	Involved in the study
☒	☐ ChIP-seq
☐	☒ Flow cytometry
☒	☐ MRI-based neuroimaging

Antibodies

Antibodies used

23-parameter flow cytometric analysis was perform on fresh EDTA whole blood, PBMCs, and mononuclear cells (10^6 cells) derived from LN biopsies, BAL, and lung. Immunophenotyping was performed using anti-human monoclonal antibodies (mAbs), which we (PMIDs: 33278358, 29045906, 26551680) and others, including databases maintained by the NHP Reagent Resource (MassBiologics), have shown as being cross-reactive in RMs. A panel of the following mAbs was used for the longitudinal phenotyping of innate immune cells in whole blood (500 µL), and mononuclear cells (10^6 cells) derived from LN biopsies, BAL, and lung from Cohort 1 and baricitinib-treated RMs: anti-CD20-BB700 (clone 2H7; 2.5uL; cat. # 745889), anti-Ki-67-BV480 (clone B56; 5uL; cat. # 566109), anti-CD14-BV605 (clone M5E2; 2.5uL; cat. # 564054), anti-CD56-BV711 (clone B159; 2.5uL; cat. # 740781), anti-CD115-BV750 (clone 9-4D2-1E4; 2.5uL; cat. # 747093), anti-CD3-BUV395 (clone SP34-2; 2.5uL; cat. # 564117), anti-CD8-BUV496 (clone RPA-T8; 2.5uL; cat. # 612942), anti-CD45-BUV563 (clone D058-1283; 2.5uL; cat. # 741414), anti-CCR2-BUV661 (clone LS132.1D9; 2.5uL; cat. # 750472), anti-CD16-BUV737 (clone 3G8; 2.5uL; cat. # 564434), anti-CD69-BUV805 (clone FN50; 5uL; cat. # 748763), and Fixable Viability Stain 700 (2uL; cat. # 564997) all from BD Biosciences; anti-CD38-FITC (clone AT1; 5uL; cat. # 60131FI) from STEMCELL Technologies; anti-CD161-BV421 (clone HP-3G10; 5uL; cat. # 339914), anti-HLA-DR-BV650 (clone L243; 5uL; cat. # 307650), anti-CD11c-BV785 (clone 3.9; 5uL; cat. # 301644), anti-CD11b-PE (clone ICRF44; 2.5uL; cat. # 301306), and anti-CD123-APC-Fire750 (clone 315; 2.5uL; cat. # 306042) all from Biolegend; anti-GranzymeB-PE-TexasRed (clone GB11; 2.5uL; cat. # GRB17) from Thermo Fisher; anti-CD66abce-PE-Vio770 (clone TET2; 1uL; cat. # 130-119-849) from Miltenyi Biotec; and anti-CD27-PE-Cy5 (clone 1A4CD27; 2.5uL; cat. # 6607107) and anti-NKG2A-APC (clone Z199; 5uL; cat. # A60797) from Beckman Coulter (Supplementary Fig. 4).

For Cohort 2 animals, a different panel of the following mAbs was used for the longitudinal phenotyping of innate immune cells in whole blood (500 µL), as described in (90), and mononuclear cells (2x106 cells) derived from LN biopsies, BAL, and lung: anti-CD20-BB700 (clone 2H7; 2.5 µL; cat. # 745889), anti-CD11b-BV421 (clone ICRFF44; 2.5 µL; cat. # 562632), anti-Ki-67-BV480 (clone B56; 5 µL; cat. # 566109), anti-CD14-BV605 (clone M5E2; 2.5 µL; cat. # 564054), anti-CD56-BV711 (clone B159; 2.5 µL; cat. # 740781), anti-CD163-BV750 (clone GHI/61; 2.5 µL; cat. # 747185), anti-CD3-BUV395 (clone SP34-2; 2.5 µL; cat. # 564117), anti-CD8-BUV496 (clone RPA-T8; 2.5 µL; cat. # 612942), anti-CD45-BUV563 (clone D058-1283; 2.5 µL; cat. # 741414), anti-CCR2-BUV661 (clone LS132.1D9; 2.5 µL; cat. # 750472), anti-CD16-BUV737 (clone 3G8; 2.5 µL; cat. # 564434), anti-CD101-BUV805 (clone V7.1; 2.5 µL; cat. # 749163), anti-CD169-PE (clone 7-239; 2.5 µL; cat. # 565248), and anti-CD206-PE-Cy5 (clone 19.2; 20 µL; cat. # 551136) and Fixable Viability Stain 700 (2 µL; cat. # 564997) all from BD Biosciences; anti-ACE2-AF488 (clone Polyclonal; 5 µL; cat. # FAB9332G-100UG) from R & D; anti-HLA-DR-BV650 (clone L243; 5 µL; cat. # 307650), anti-CD11c-BV785 (clone 3.9; 5 µL; cat. # 301644), and anti-CD123-APC-Fire750 (clone 315; 2.5 µL; cat. # 306042) all from Biolegend; anti-GranzymeB-PE-TexasRed (clone GB11; 2.5 µL; cat. # GRB17) from Thermo Fisher; anti-CD66abce-PE-Vio770 (clone TET2; 1 µL; cat. # 130-119-849) from Miltenyi Biotec; anti-NKG2A-APC (clone Z199; 5 µL; cat. # A60797) from Beckman Coulter. mAbs for chemokine receptors (i.e. CCR2) were incubated at 37C° for 15 min, and cells were fixed and permeabilized at room temperature for 15 min with Fixation/Permeabilization Solution Kit (BD Biosciences; cat. #554714).

Validation

All antibodies (anti-human) are validated and annotated as human reactive by the manufacturers on their associated catalog pages (they typically do not test/report for RM reactivity or are listed as potentially macaque reactive). All mAbs used in this study were either previously verified as rhesus reactive in our previous studies (PMIDs: 33278358, 29045906, 26551680) or were independently verified as rhesus-reactive via databases maintained by the NHP Reagent Resource. All mAbs have previously been internally validated via FMO tests.

Animals and other research organisms

Policy information about studies involving animals; ARRIVE guidelines recommended for reporting animal research, and Sex and Gender in Research

Laboratory animals

Cohort 1 (n=4) and baricitinib cohort (n=4): 4 female and 4 male Indian rhesus macaques (RMs; Macaca mulatta), all housed at the

	Yerkes National Primate Research Center (YNPRC) at Atlanta, Ga, were included in this study. Animals were between 141 and 204 months old at time of infection. Cohort 2: 6 additional rhesus macaques (2 females and 4 males) were recruited in the study subsequently (mean age 10.5 years old; range 6-19.5 years old),
Wild animals	Study did not involve wild animals.
Reporting on sex	The 8 animals under IACUC permit PROTO202000035 were age and sex matched between untreated control and baricitinib-treated experimental arms, with 2 females and 2 males assigned to each respective arm. Cohort 2 (IACUC permit PROTO202100003) was comprised of 2 females and 4 males, all of which served as untreated controls. Efforts to include equal numbers of females and males in Cohort 2 were made. However, female macaques were limited at the time of Cohort 2 animal assignment due to breeding demands. In total, between Cohort 1 and 2, 4 female and 6 male untreated controls were included in this study.
Field-collected samples	Study did not involve field-collected samples.
Ethics oversight	All animal procedures were performed in line with institutional regulations and guidelines set forth by the NIH's Guide for the Care and Use of Laboratory Animals, 8th edition, and were conducted under anesthesia with appropriate follow-up pain management to minimize animal suffering. All animal experimentation was reviewed and approved by Emory University's Institutional Animal Care and Use Committee (IACUC) under permit PROTO202000035 (Cohort 1) and PROTO202100003 (Cohort 2).

Note that full information on the approval of the study protocol must also be provided in the manuscript.

Flow Cytometry

Plots

Confirm that:

- [x] The axis labels state the marker and fluorochrome used (e.g. CD4-FITC).
- [x] The axis scales are clearly visible. Include numbers along axes only for bottom left plot of group (a 'group' is an analysis of identical markers).
- [x] All plots are contour plots with outliers or pseudocolor plots.
- [x] A numerical value for number of cells or percentage (with statistics) is provided.

Methodology

Sample preparation	Phenotypic analyses were performed on cellular suspensions derived from fresh tissue. Peripheral whole blood (PB) was collected from the femoral vein in sodium citrate, serum separation, and EDTA tubes from which plasma or serum was separated by centrifugation within 1 hour of phlebotomy. Peripheral blood (PB) collected in EDTA was used for complete blood counts, measurement of neutrophil extracellular traps (NET) activity, whole blood staining, and PBMC isolation and staining. Following centrifugation and the separation of plasma, 500uL of the remaining fraction of blood was lysed with ACK lysis buffer, pelleted via centrifugation, washed twice with PBS, and stained with the panels of mAbs used for the longitudinal phenotyping of innate immune cells. Peripheral blood mononuclear cells (PBMCs) were also isolated from the blood fraction remaining after plasma removal using a Ficoll-Paque Premium density gradient (GE Healthcare), and washed with R-10 media that was composed of RPMI 1640 (Corning) supplemented with 10% heat-inactivated fetal bovine serum (FBS), 100 IU/mL penicillin, 100 µg/mL streptomycin, and 200 mM L-glutamine (GeminiBio). To collect BAL, a fiberoptic bronchoscope (Olympus BF-XP190 EVIS EXERA III ULTRA SLM BRNCH and BF-P190 EVIS EXERA 4.1mm) was manipulated into the trachea, directed into the primary bronchus, and secured into a distal subsegmental bronchus upon which 35-50 mL of normal saline (0.9% NaCl) was administered into the bronchus and re-aspirated to obtain a minimum of 20ml of lavage fluid. BAL was filtered through a 70µm cell strainer and then centrifuged. BAL fluid supernatant was removed and pelleted BAL cells were resuspended in R10 and then stained fresh with both the innate immune cell and T-cell phenotyping flow panels. Hilar LN biopsies and the bilateral upper portions of the caudal (lower) lung lobes were collected at necropsy. Hilar LNs were sectioned using blunt, micro-dissection scissors, mechanically disrupted through a 70µm cell strainer, washed with R-10 media, and stained fresh with innate immune cell and T-cell phenotyping panels. Lung tissue was injected with digestion media containing 0.4mg/mL DNase I (StemCell Technologies), 2.5 mg/mL of Collagenase D (Roche), and 0.2mg/mL Liberase TL Research Grade (Sigma-Aldrich) in HBSS using a blunt end needle. Next, lung tissue was cut into small pieces using blunt end scissors, incubated in digestion media at 37°C, and homogenized using a gentleMACS Dissociator, program "Lung 02_01" (Miltenyi Biotec). The resulting homogenized tissue was filtered through a 100µm BD Falcon cell strainer, washed with R-10 media, and stained fresh with innate immune cell and T cell phenotyping panels.
Instrument	FACSymphony (BD Biosciences) for phenotyping,
Software	Data collected on FACSSymphony driven by FACS DiVa software (BD Biosciences). FlowJo version 10.7 (TreeStar).
Cell population abundance	The sorted interstitial and alveolar macrophages that were used for bulk RNA-Seq from a published study (PMID: 35271686) were reported to have a >90% purity for each cell population.

Gating strategy	Mononuclear cells were defined by laying on the diagonal of FSC-H versus FSC-A, leukocytes were gated as CD45+, and granulocytes and a combined lymphocyte and monocyte population were gated from SSC-A versus FSC-A. For the innate immune cell panel, monocytes were pre-gated from the combined lymphocyte and monocyte population as live CD3-, HLA-DR+ CD20- and gated according to CD14 vs. CD16 expression and CCR2 exoression and pDCs were pre-gated as CD14-, CD123+. B cells were pre-gated as live CD3-, HLA-DR+ CD20+ while NK cells were pre-gated as live CD3-, HLA-DR- CD20-, CD8+NKG2A+ and gated according to CD16 and CD56 expression. Neutrophils were identified as CD45+CD3-CD20-CD66+ live granulocytes.

☒ Tick this box to confirm that a figure exemplifying the gating strategy is provided in the Supplementary Information.
